# A CD36-targeted aptamer-4-butyl-polyhydroxybenzophenone conjugate with pH-responsive release for liver delivery in MASLD

**DOI:** 10.1093/rb/rbag104

**Published:** 2026-06-01

**Authors:** Luyao Ren, Yuxi Qin, Hongxiao Lu, Siyu Lu, Meiying Liu, Huanhuan Zhang, Lina Guo, Guang Zhao, Yunlan Li

**Affiliations:** School of Pharmaceutical Science, Shanxi Medical University, Taiyuan 030001, China; School of Pharmaceutical Science, Shanxi Medical University, Taiyuan 030001, China; School of Pharmaceutical Science, Shanxi Medical University, Taiyuan 030001, China; School of Pharmaceutical Science, Shanxi Medical University, Taiyuan 030001, China; School of Pharmaceutical Science, Shanxi Medical University, Taiyuan 030001, China; School of Pharmaceutical Science, Shanxi Medical University, Taiyuan 030001, China; School of Pharmaceutical Science, Shanxi Medical University, Taiyuan 030001, China; School of Pharmaceutical Science, Shanxi Medical University, Taiyuan 030001, China; School of Pharmaceutical Science, Shanxi Medical University, Taiyuan 030001, China

**Keywords:** MASLD, aptamer-drug conjugate, CD36, targeted delivery platform, 4-butyl-polyhydroxybenzophenone compounds

## Abstract

Metabolic dysfunction-associated steatotic liver disease (MASLD) is a widespread chronic liver disease worldwide, making efficient targeted delivery systems essential to alleviate its socioeconomic burden. Aptamers are short, single-stranded DNA or RNA oligonucleotides that bind to specific target antigens with high affinity and specificity. Compared with antibody-based therapies, aptamer-based therapies offer the advantages of small size, facile synthesis and low immunogenicity. Herein, we report a novel fatty acid translocase CD36-targeted aptamer−drug conjugate (ApDC), the NAFLD01 aptamer conjugated with 4-(4-(tert-butyl)benzoyl)-2,3-dihydroxyphenyl furan-2-carboxylate (SF), termed ASC, as a targeted delivery platform for MASLD. Results demonstrated that SF significantly reduced body weight, attenuated liver injury and ameliorated lipid accumulation in high-fat diet (HFD)-induced MASLD mice. ASC effectively reduced lipid droplet formation and hepatic injury biomarker levels, while improving antioxidant enzyme levels in MASLD cell models *in vitro*. A key advantage of ASC is its capacity for targeted SF delivery, which potentially reduces off-target exposure of SF to non-target organs. ASC retained the intrinsic properties of the NAFLD01 aptamer, including high binding affinity and efficient internalization by target cells. Additionally, the acid-labile hydrazone bond in ASC was cleaved in an acidic lysosomal environment. In conclusion, ASC is a promising ApDC delivery platform for MASLD.

## Graphical abstract

**Graphical Abstract:**
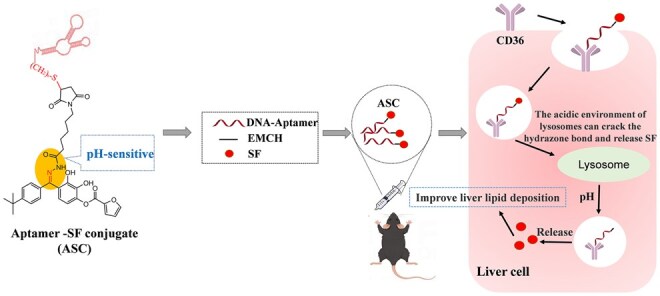

## Introduction

Metabolic dysfunction-associated steatotic liver disease (MASLD), the updated nomenclature for nonalcoholic fatty liver disease (NAFLD), is now recognized as a leading metabolic liver disorder. Closely associated with obesity, type 2 diabetes, dyslipidemia and hypertension, MASLD affects about 25–30% of individuals worldwide [1, 2]. The disease spans a broad pathological spectrum, ranging from nonalcoholic fatty liver (NAFL) to metabolic dysfunction-associated steatohepatitis (MASH) and may ultimately lead to cirrhosis or hepatocellular carcinoma (HCC) [3, 4]. Diet and exercise have demonstrated efficacy in the treatment of MASLD; however, long-term patient compliance is poor. In clinical practice, insulin-sensitizing and lipid-lowering agents are commonly prescribed; however, their therapeutic efficacy, specificity and safety remain suboptimal [5]. Consequently, the formulation of efficient and specific therapy approaches for MASLD is an immediate clinical necessity.

Liver-targeted medication delivery methods are regarded as highly effective approaches for treating liver diseases, as they can prolong the circulation time of drugs and precisely deliver medications to the liver while minimizing side effects [6]. Aptamers are short single-stranded nucleic acid ligands generated through systematic evolution of ligands by exponential enrichment (SELEX). Their folded three-dimensional shapes let them bind to certain molecular targets [7–9]. Aptamers are often called “chemical antibodies” due to their affinity and specificity being analogous to those of antibodies [10]. Aptamers have numerous advantages compared to antibodies, including smaller molecular size, straightforward chemical manufacturing, low immunogenicity and flexible modification [11]. Consequently, aptamers are highly suitable for targeted drug delivery, diagnostic tools and molecular therapy [12].

CD36 is a transmembrane glycoprotein that primarily binds to oxidized low-density lipoprotein (LDL) and facilitates fatty acid entry into hepatocytes [13, 14]. In MASLD patients, hepatic CD36 upregulation is closely linked to excessive lipid deposition and may promote the transition from simple steatosis to MASH [15]. Recently, Pu *et al.* found that NAFLD01 is a CD36-specific aptamer that can specifically recognize MASLD cells, with a dissociation constant (Kd) of 21.5 ± 5.6 nmol/L [16]. Our previous research synthesized a series of 4-butyl-polyhydroxybenzophenone compounds, which can markedly reduce hepatic fat buildup and liver damage in MASLD mice, as well as their antioxidant stress ability [17]. However, the lack of tissue specificity may limit their therapeutic efficiency and increase the risk of off-target effects.

Therefore, we developed a novel CD36-targeted ApDC by conjugating the NAFLD01 aptamer with the 4-butyl-polyhydroxybenzophenone derivative SF via a pH-sensitive hydrazone bond. This system is designed to enhance the liver targeting and cellular uptake of SF, aiming to establish a novel aptamer-based liver-targeted delivery platform for MASLD.

## Materials and methods

### Reagents

HepG2, L-02, MCF-7 and GES-1 cell lines were given by Procell Life Science & Technology Co., Ltd (Wuhan, China). The rabbit monoclonal antibodies against CD36 (A5792) and β-actin (A19667) were obtained from ABclonal Technology (Wuhan, China). Fetal bovine serum, DMEM and RPMI-1640 were provided by Boster Biological Technology Co., Ltd. Fenofibrate, dimethyl sulfoxide (DMSO), Oil Red O Staining Kit (G1262), Nucleic acid dye Gelred (41003), MTT (M1020) and yeast tRNA (T8630) were acquired from Beijing Solarbio Technology. Wuhan Servicebio Co., Ltd was the supplier of BSA (GC305010), glucose (GC205003) and MgCl_2_ (GC102002). LysoTracker Green lysosomal probe (C1047S) and TCEP were supplied by Beyotime Biotechnology Co., Ltd (Nanjing, Jiangsu, China). N-ε-maleimidocaproic acid hydrazide (EMCH, 81186-33-6) was purchased from Jiangsu Aikang Co., Ltd. AST kit (C010-2), TC kit (A111-1-1), ALT kit (C009-2-1), TG kit (A110-1), GSH kit (A006-2-1), HDL kit (A112-1-1), LDH kit (A020-2-2) and LDL kit (A113-1-1) were acquired from Nanjing Jiancheng Bioengineering. Enzyme-Link Biotechnology Co., Ltd supplied the ELISA kits for IL-6, IL-1β and TNF-α. Butylpolyhydroxybenzophenone compounds were synthesized in our laboratory with a purity greater than 95%. Aptamers were synthesized by Huzhou Seahorse Biotechnology Co., Ltd. Supplementary Table S1 lists the nucleic acid aptamer sequence used in this study.

### Cell culture and selection of models

HepG2 and L-02 cells were selected as hepatocyte models related to MASLD because they have active lipid metabolism and are widely utilized in MASLD-related research [18, 19]. In contrast, MCF-7 and GES-1 were selected as negative control cell lines, as they are of non-liver origin and show low CD36 expression. These characteristics make them suitable for evaluating CD36-mediated targeting specificity.

HepG2, MCF-7 and GES-1 cells were grown in DMEM, while L-02 cells were grown in RPMI-1640. The culture media contained 10% fetal bovine serum and 1% penicillin–streptomycin. All cells were kept at 37°C in a 95% humidity incubator with 5% CO_2_. The cell lines were negative for mycoplasma. Palmitic acid (PA) was conjugated with BSA at a molar ratio of 13:1 (PA: BSA). Cells were induced with 500 μmol/L PA for 24 h for the establishment of the MASLD *in vitro* model.

### Bioinformatics analysis

MASLD-related candidate genes were collected from OMIM, DrugBank and GeneCards. After data integration, the integrated targets were submitted to STRING for PPI network generation, and only interactions with a confidence score >0.9 were retained. The network was then visualized and analyzed in Cytoscape 3.8.2, where hub targets were identified using node degree. Functional enrichment of the core targets was subsequently performed using the Metascape platform, with emphasis on GO biological process analysis.

### Molecular docking

Structural files for PPARG, PPARA and CD36 were downloaded from the RCSB Protein Data Bank and processed with SYBYL 2.0 prior to docking. The docking simulations were performed in SYBYL-X with the Surflex-Dock protocol. Docking scores were used to reflect the binding strength between compounds and predicted targets, with scores ≥5.00 regarded as high-affinity binding [20, 21].

The six candidate compounds listed in Table 1 were selected from a series of 4-butyl-polyhydroxybenzophenone derivatives previously synthesized and reported by our group (Patent No. CN202010178813.5). In previous structure–activity relationship and pharmacological studies, several compounds from this series demonstrated hepatoprotective and lipid-regulating activities in MASLD models [17, 22]. Based on these findings, six compounds with favorable bioactivity and representative structural characteristics were selected as candidate molecules. Subsequently, molecular docking analysis was performed to compare their binding affinities toward key MASLD-associated targets and identify the compound exhibiting the highest predicted binding potential for further experimental investigation.

**Table 1:** Docking scores for 4-butyl-polyhydroxybenzophenone compounds.

Compd.	Structure	Total Score		
		PPARG (PDB:3DZY)	PPARA (PDB:3SP6)	CD36 (PDB:5LGD)
SF	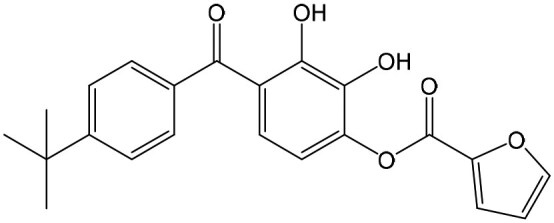	6.0815	7.0523	8.5512
Y9	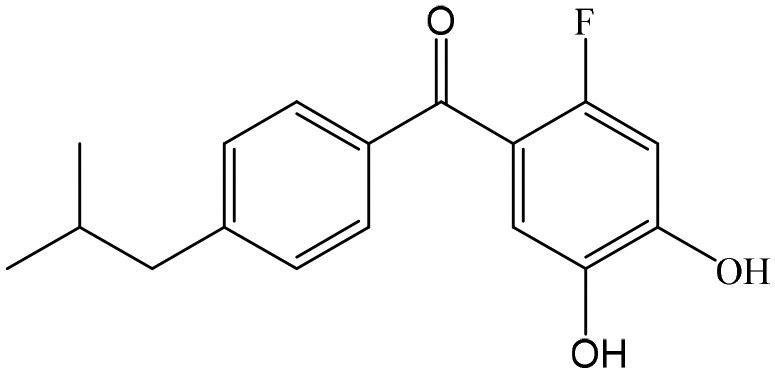	4.1070	6.5084	7.6047
S2	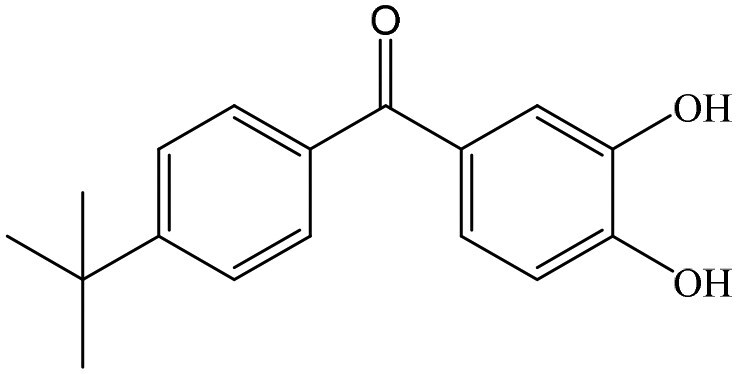	4.0601	5.3968	5.4512
D2	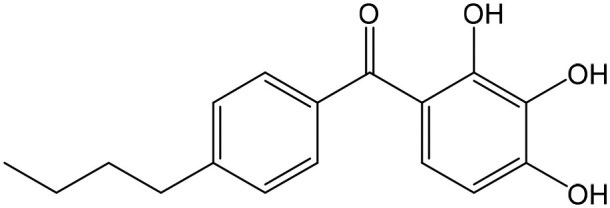	6.3244	6.3313	6.4254
S9	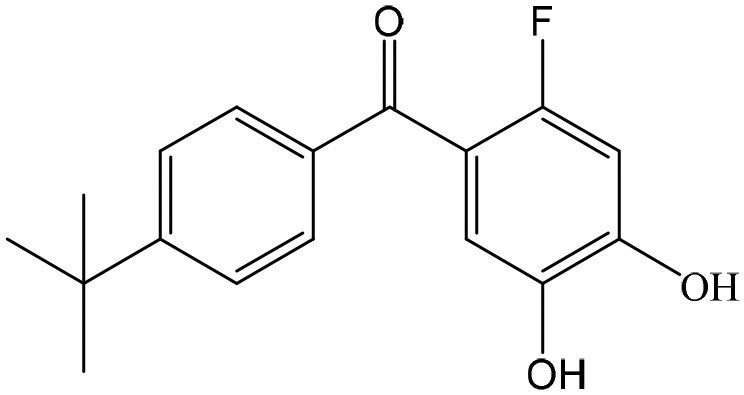	5.8325	5.7281	5.6286
WM	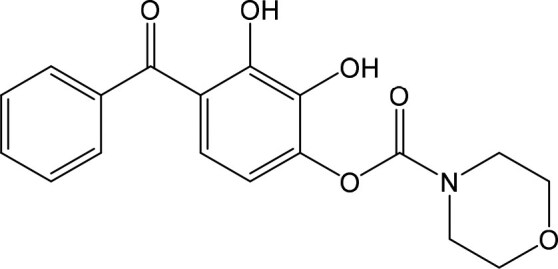	4.9431	5.9279	5.7447

### Western blot and RNA interference assay

Cell lysates were prepared with RIPA buffer and assayed by BCA. Protein samples were run on SDS-PAGE and transferred to nitrocellulose membranes (Millipore). Membranes were blocked with 5% skimmed milk for 1 h at room temperature, incubated with anti-CD36 (1:1000) and anti-β-actin (1:10 000) overnight at 4°C and then treated with HRP-linked secondary antibodies for 1 h at room temperature. Bands were detected by ECL.

After being plated in 6-well plates, HepG2 cells were given 24 h to reach 70–80% confluence. Next, siRNA-mate Plus reagent (7.5 μL per well) was used to treat the cells with CD36 siRNA or negative control siRNA at 75 pmol per well. Following a 24-h transfection period, a new medium containing 500 μmol/L PA was added to the culture media, and the process continued for an additional 24 h. The silencing effect of CD36 was examined by western blotting. CD36 siRNA, negative control siRNA and siRNA-mate Plus reagent were purchased from GenePharma.

### Immunofluorescence staining

Cells were seeded in 6-well plates, and L-02 and HepG2 cells were treated with 500 μmol/L PA for 24 h before staining. After fixation with 4% paraformaldehyde, cells were permeabilized, blocked with 5% BSA and incubated with the primary antibody overnight at 4°C. All subsequent steps were performed in the dark. Cells were then incubated with secondary antibody, stained with DAPI, mounted with anti-fade medium and imaged by fluorescence microscopy. ImageJ was used for image analysis.

### Animal grouping and modeling

Male ICR mice of 6-week-old were provided by the Laboratory Animal Resource Center of Shanxi Medical University. All animal procedures were conducted after approval by the Animal Ethics Committee of the Laboratory Animal Research Center, Shanxi Medical University, Taiyuan, China (No. 2024LL097). The mice were maintained in a controlled environment at 22 ± 2°C under a 12-h light/12-h dark cycle, with free access to food and water. A total of 36 mice were randomly assigned to 6 groups, 6 mice per group: control, HFD, low-dose SF (10 mg/kg), medium-dose SF (20 mg/kg), high-dose SF (30 mg/kg) and fenofibrate positive control (30 mg/kg). Fenofibrate is a clinically approved lipid‑regulating agent with potent lipid-lowering, antioxidant, anti‑inflammatory and anti‑fibrotic activities. It was thus selected as the positive control [23]. The control group was maintained on standard chow, 5 g/d, while all remaining groups received an equal amount of 60% high-fat diet for 8 weeks to induce MASLD. After 8 weeks of model induction, mice in the treatment groups continued to be fed the 60% HFD and received corresponding interventions, while the model group continued HFD feeding without drug treatment. Treatment was then given by daily oral gavage for 4 weeks. Body weight and general condition were monitored. At the end of the experiment, mice were anesthetized with isoflurane, euthanized and sampled for analysis.

### Animal biochemical and histological analysis

Mouse liver tissues were fixed with 4% paraformaldehyde, processed for paraffin embedding and stained with hematoxylin and eosin (H&E) to evaluate hepatic steatosis. Frozen liver sections were stained with Oil Red O and counterstained with hematoxylin to assess hepatic lipid deposition. Whole blood was left at room temperature and centrifuged to collect serum, while liver tissues were homogenized on ice and centrifuged to obtain supernatants. Serum and liver supernatants were assayed using commercial kits for lipid-related indices, including triglycerides (TG), high-density lipoprotein (HDL), total cholesterol (TC) and low-density lipoprotein (LDL); liver injury markers, including alanine aminotransferase (ALT), aspartate aminotransferase (AST) and lactate dehydrogenase (LDH); inflammatory cytokines, including interleukin-6 (IL-6), interleukin-1β (IL-1β) and tumor necrosis factor-α (TNF-α); and glutathione (GSH).

### Synthesis of ASC

Briefly, SF (15 mg) and N-ε-maleimidocaproic acid hydrazide (EMCH) (27 mg) were dissolved in anhydrous ethanol (4 mL), followed by the addition of glacial acetic acid. The mixture underwent reflux at 80°C for 24 h, with reaction progress tracked via thin-layer chromatography (TLC). Post-reaction, products were isolated and purified by preparative TLC to afford (6-maleimidocaproyl) hydrazone of SF (9 mg, 39.1% yield).

Thiolated aptamers were reduced with TCEP (100 mmol/L, pH 6.5) for 1 h at room temperature to expose sulfhydryl groups. After heating at 70°C for 10 min and cooling on ice, the aptamer was reacted with the SF hydrazone derivative in dimethylformamide (DMF) overnight at 4°C. Excess small molecules were removed by dialysis, and ASC was purified on an Agilent 1260 Infinity HPLC system with a Phenomenex Clarity 3 μm Oligo-RP column using methanol/50 mM triethylammonium acetate (TEAA, pH 7.5; 20:80, v/v) at 1.0 mL/min, with detection at 260 nm.

### Determination of biostability of ASC in serum

ASC and nucleic acid aptamers were individually prepared in RPMI-1640 medium enriched with 10% FBS at a final concentration of 2.5 μmol/L. The samples were incubated at 37°C for durations of 0, 2, 4, 12, 24 and 48 h. Subsequently, 10 μL of each sample was applied to a 3% agarose gel and visualized using a gel imaging system.

### pH-dependent drug release kinetics

SF release from ASC at different pH values was evaluated by dialysis. ASC was dissolved in PBS buffer at different pH values (5.0, 6.5 and 7.4) and incubated at 37°C with shaking at 100 rpm. Samples were taken at 0, 1, 4, 8, 12, 24, 36 and 48 h, analyzed by HPLC and release curves were constructed to calculate the cumulative release amount of free SF (methodological details are shown in Supplementary Figures S1 and S2; Supplementary Tables S2 and S3).

### Study of the specificity and binding ability of ASC

The binding abilities of NAFLD01 and ASC were assessed using flow cytometry. Cells (5 × 10^5^) were incubated in 400 μL of binding buffer at 37°C for 2 h, followed by three washes with washing buffer and finally suspended in 400 μL of washing buffer before FACS analysis. Data were analyzed via FlowJo software. Measure the background signal using cells that have not been treated.

Washing buffer: 5 mmol/L MgCl_2_ and 4.5 g/L glucose in DPBS. Binding buffer: 5 mmol/L MgCl_2_, 4.5 g/L glucose, 0.1 mg/mL yeast tRNA and 1 mg/mL BSA in DPBS.

For flow cytometric analysis, cells were first gated on FSC and SSC to exclude debris and select the main cell population. Doublets were then excluded by plotting FSC-A versus FSC-H to retain only single-cell events. Fluorescence intensity was analyzed within the gated single-cell population. At least 10 000 events were collected per sample. Each experiment included three biological replicates (*n* = 3).

### Cellular uptake analysis

In confocal culture dishes, 1 × 10^5^ cells of L-02, HepG2, GES-1 and MCF-7 were seeded and cultured for 24 h. L-02 and HepG2 cells were subsequently induced with 500 μmol/L PA for 24 h. The four cell groups were incubated with 250 nmol/L Cy5-labeled ASC at 37°C, 5% CO_2_, for 2 h and then, 100 nmol/L LysoTracker Green lysosomal probe was added and incubated for 1 h. After PBS washing, cells were imaged by confocal microscopy with a 100× oil objective.

To observe the effect of CD36 knockdown on ASC internalization, HepG2 cells were transfected with siCD36 or siNC for 24 h, followed by treatment with 500 μmol/L PA for another 24 h. Subsequently, cells were incubated with 250 nmol/L Cy5-labeled ASC for 2 h. Images were acquired using a high-content imaging system. Bright-field images were used to visualize cell morphology, and the Cy5 channel (red fluorescence) was applied to record the intracellular uptake of ASC.

### *In vivo* imaging

MASLD mice received intravenous injection of Cy5-ASC or Cy5-CASC, 100 μL per mouse at 50 μmol/L. After anesthesia, mice were imaged using an *in vivo* imaging system.

Fluorescence imaging was performed using an IVIS Lumina XRMS III system (PerkinElmer). Images were acquired using Cy5 excitation and emission filters with automatic exposure, a binning factor of 8, an f/stop of 2 and all parameters were kept consistent across groups. Regions of interest (ROI) were manually drawn over the liver area based on anatomical location, with consistent size and position across all images and fluorescence signals were quantified as average radiant efficiency (p/sec/cm^2^/sr)/(μW/cm^2^). Background signals were subtracted prior to analysis. For visualization, all images were displayed using a uniform color scale to allow direct comparison between groups. For *ex vivo* imaging, major organs were collected and imaged under identical settings, and fluorescence distribution was analyzed accordingly. Each group included three independent mice (*n* = 3).

### Measurement of SF content inside and outside L-02 cells

L-02 cells were treated with 500 μmol/L PA for 24 h, followed by SF or ASC for 4 h. Cell pellets and culture media were collected separately. Pellets were washed with PBS, extracted with 200 μL methanol, centrifuged, dried and redissolved in 30 μL methanol. Culture media were mixed with an equal volume of methanol, sonicated and centrifuged. SF was quantified by HPLC on an Agilent 1260 Infinity system with an Agilent C18 column. Detection was performed at 255 nm with a flow rate of 1.0 mL/min, column temperature of 30°C and injection volume of 20 μL.

Cells were treated with SF and ASC for 4 h, and then the samples of each group were treated according to the procedure. Samples of 20 μL were injected for HPLC analysis. Then, at 0.5, 1, 2, 4 and 6 h, the content of SF in ASC and SF groups was detected to evaluate the content entering cells at different time points.

### *In vitro* toxicity and cell protective activity assay of ASC

L-02 cells were plated in 96-well plates at about 1 × 10^4^ cells/well and cultured for 24 h. To establish the MASLD cell model, cells were exposed to 500 μmol/L PA for 24 h and then treated with different concentrations of SF or ASC. After another 24 h, absorbance was read at 490 nm and the EC_50_ value was calculated using GraphPad Prism 8.

### Intracellular biochemical marker assays

Cells were collected and centrifuged at 1000 rpm for 10 min. The supernatant was discarded, and the cell pellets were resuspended in PBS and homogenized on ice. After BCA-based protein quantification, TC, TG, AST, ALT, LDH and GSH were measured according to the kit instructions.

### Statistical analysis

Statistical analyses were carried out in GraphPad Prism 8. Data normality was checked with the Shapiro–Wilk test. For two-group comparisons, an unpaired Student’s t-test was applied, whereas datasets involving more than two groups were analyzed by one-way ANOVA with Tukey’s test for pairwise *post hoc* comparisons. Results are expressed as mean ± SD. Differences with *P *< 0.05 were considered significant.

## Results

### PPARG, PPARA and CD36 are the core targets of MASLD

GeneCards and DrugBank yielded 1465 gene targets related to MASLD. Among them, the 300 targets with the highest relevance scores were used to build the interaction network. The MASLD-related PPI network was examined in Cytoscape, and hub targets were selected according to node degree. Ultimately, we identified 10 key hub targets. Among them, PPARA, PPARG and CD36 were considered essential nodes (Supplementary Figure S3A). In the Gene Ontology (GO) biological process analysis, the core targets were mainly linked to small-molecule metabolic regulation, lipid homeostasis and storage and lipid biosynthesis (Supplementary Figure S3B).

### SF is the best candidate compound targeting PPARG, PPARA and CD36

**Figure 1:**
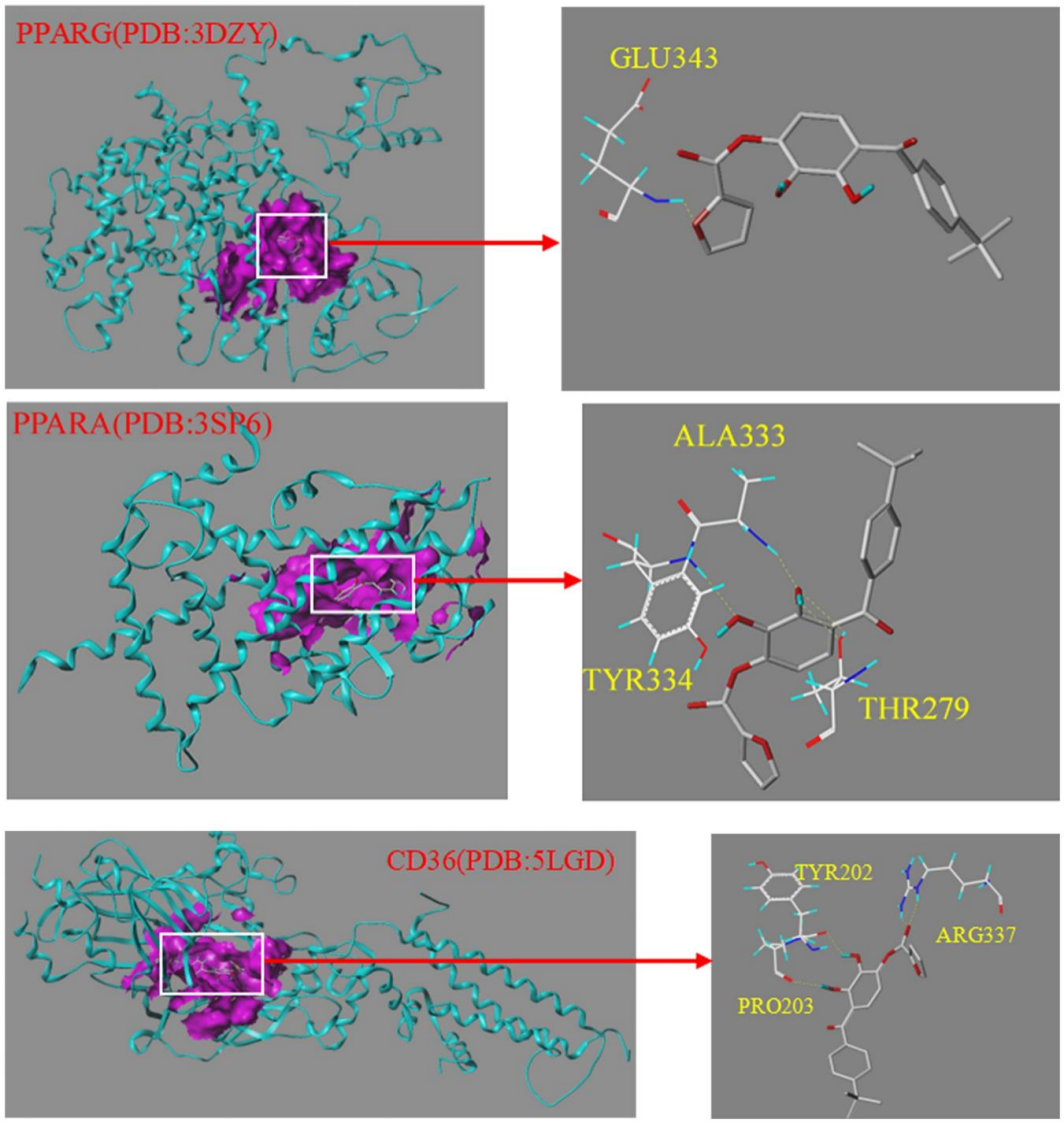
Docking results of SF with PPARG (PDB:3DZY), PPARA (PDB:3SP6) and CD36 (PDB:5LGD).

Molecular docking analysis between the 4-butyl-polyhydroxybenzophenone derivatives and the three targets, PPARG, PPARA and CD36, showed that SF was the best candidate compound targeting PPARG, PPARA and CD36, and its docking scores with PPARG, PPARA and CD36 were 6.0815, 7.0523 and 8.5512, respectively (Table 1). Oxygen atoms of the SF furan ring interacted with GLU343 on PPARG through hydrogen bonds, and hydroxyl groups on the benzene ring interacted with PPARA through hydrogen bonds at ALA333, TYR334 and THR279 sites, among which GLU343 and THR279 are located at the active binding sites of PPARG and PPARA (Figure 1). Although PPARG and PPARA showed favorable docking results, they are intracellular nuclear receptors and are not suitable for aptamer-mediated targeting. In contrast, CD36 is a membrane-localized receptor, making it more accessible for ligand recognition and internalization. Therefore, CD36 is an ideal target for the ApDC delivery system developed in this study.

The primary objective of molecular docking was to compare the relative binding affinities of candidate compounds during the preliminary virtual screening process. In subsequent *in vitro* and *in vivo* experiments, SF was employed in its intact structure; accordingly, this original structure was adopted for molecular docking to maintain consistency between computational screening and subsequent biological validation. The docking analysis showed that intact SF could establish hydrogen-bond interactions with key residues in CD36, PPARG and PPARA, suggesting a potential binding capacity. Therefore, SF was selected for further investigation in this study.

### SF reduces liver lipid deposition and injury *in vivo*

**Figure 2:**
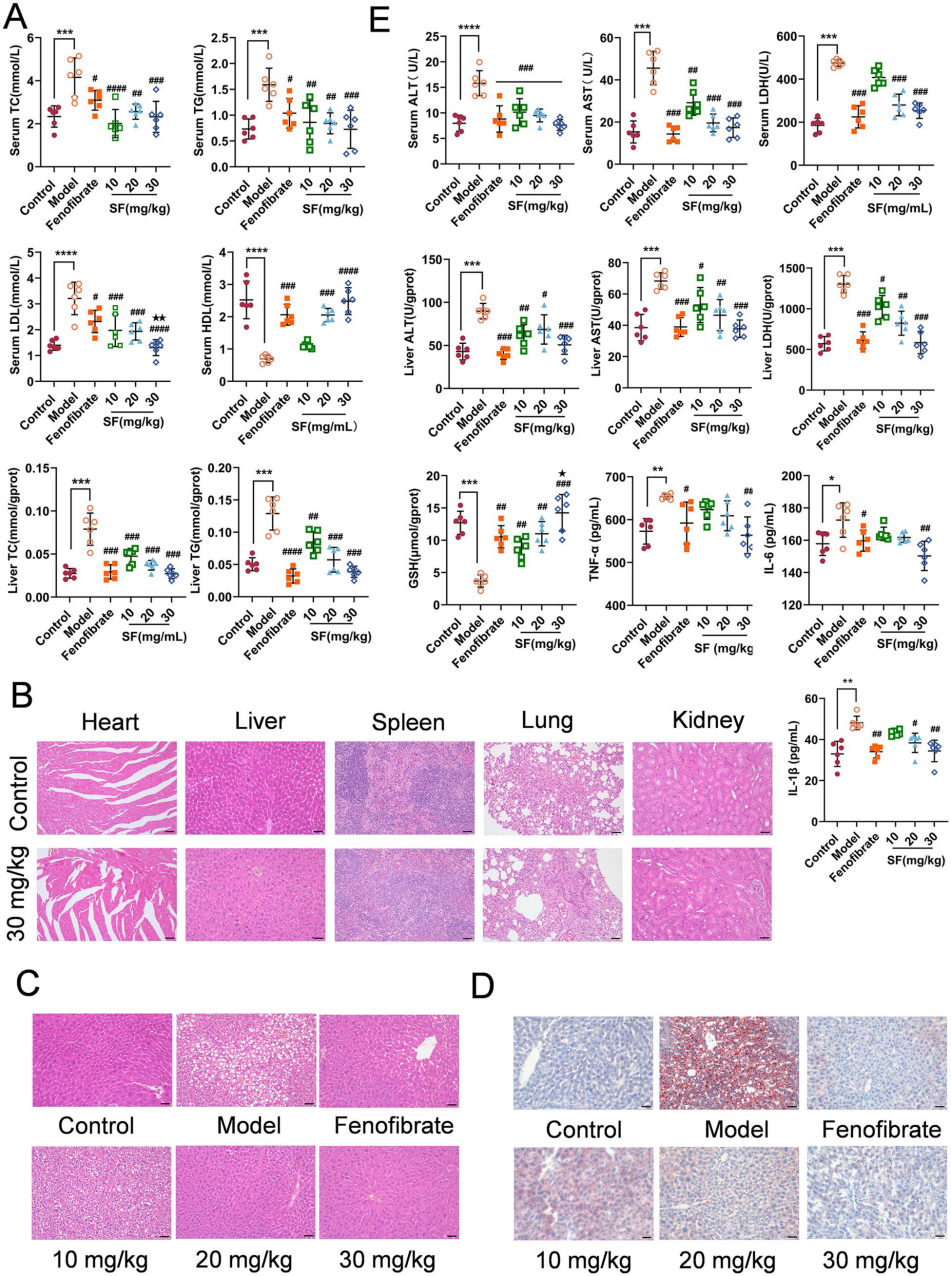
SF mitigates HFD-induced hepatic steatosis and injury. (**A**) Detection of lipid biochemical indexes in serum and liver of MASLD mice. (**B**) Representative H&E images of major organs from control and high-dose SF-treated mice. (**C**) Liver H&E staining. (**D**) Hepatic Oil Red O staining. (**E**) Liver AST, ALT, GSH and LDH levels and serum levels of LDH, AST, ALT, TNF-α, IL-6 and IL-1β. Data are presented as mean ± SD (*n* = 6). *P < 0.05, ^**^P < 0.01, ^***^P < 0.001, ^****^P < 0.0001 vs. control; ^#^*P *< 0.05, ^##^*P *< 0.01, ^###^*P *< 0.001, ^####^*P *< 0.0001 vs. model; ^★^*P *< 0.05 vs. fenofibrate.

After 12 weeks of HFD feeding, model mice showed higher body weight, liver coefficient, hepatic TG and TC contents and LDL levels, together with lower HDL levels than control mice. These changes indicated successful induction of hepatic lipid deposition and liver injury by HFD (Table 2, Figure 2A). Following administration of SF at different concentrations, the body weight, liver coefficient and lipid content in the liver and serum showed a dose-dependent significant reduction (Table 2, Figure 2A). No evident histopathological injury was observed in the liver, heart, spleen, lung or kidney after high-dose SF treatment, as shown by H&E staining (Figure 2B). In the model group, H&E staining revealed severely disrupted hepatic lobule structure, disordered hepatocyte arrangement, massive fat vacuoles, nuclei displaced to the cell periphery by lipid droplets and obvious inflammatory cell infiltration in the liver. SF treatment markedly ameliorated liver injury in MASLD mice, significantly reduced fatty vacuoles and inflammatory infiltration and nearly normalized liver morphology (Figure 2C). Significant buildup of red lipid droplets was found in the liver tissues of the model group using Oil Red O staining. Hepatic lipid droplets were markedly reduced in both number and size in the medium- and high-dose SF groups, suggesting that lipid deposition in the liver decreased significantly (Figure 2D). Further, the levels of LDH, ALT and AST in the liver and serum of model mice increased, GSH content in the liver decreased and the levels of IL-6, TNF-α and IL-1β in the serum increased significantly. SF intervention significantly reversed these changes (Figure 2E), with an effect comparable to that of the positive control fenofibrate. Overall, SF significantly improved the liver damage, hepatic lipid accumulation and systemic inflammation induced by the HFD in a dose-dependent manner.

**Table 2:** Effects of SF on mouse body weight and liver index (*n* = 6).

Group	Weight (g)	Liver index (g/g)
Control	29.7 ± 1.1	0.048 ± 0.002
Model	46.0 ± 3.3***	0.053 ± 0.002***
Fenofibrate (30 mg/kg)	29.9 ± 1.3^##^	0.048 ± 0.004^###^
SF (10 mg/kg)	32.8 ± 8.8^##^	0.044 ± 0.005^###^
SF (20 mg/kg)	29.2 ± 1.3^###^	0.043 ± 0.003^###^
SF (30 mg/kg)	28.9 ± 3.2^###^	0.044 ± 0.002^###^

Data are presented as mean ± SD.

*** *P* < 0.001 vs. control,

## *P* < 0.01 and

### *P* < 0.001 vs. model.

### Synthesis of ASC

**Figure 3:**
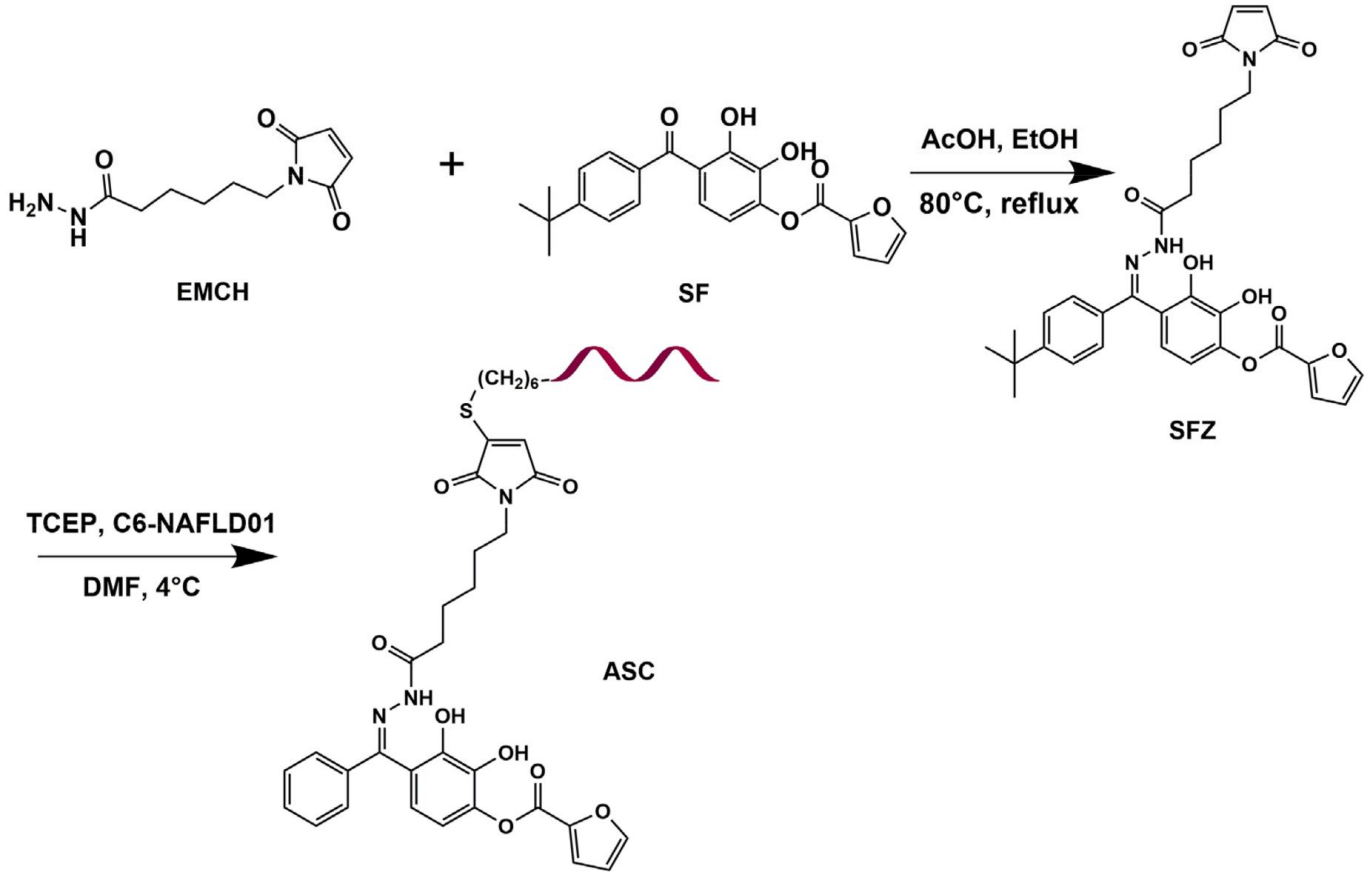
Synthetic route of ASC.

The synthesis of SF was conducted according to the literature [17]. We selected EMCH, with featuring reactive groups at both ends, as the linker molecule. A hydrazide group at the end of EMCH underwent a nucleophilic addition reaction with the carbonyl group on the benzophenone core, forming an acylhydrazone bond [24]. Meanwhile, sulfhydryl modification was introduced at the 5′ end of the DNA aptamer, reacted with the maleimide group of EMCH through a Michael addition reaction, and SF was successfully conjugated with the aptamer. The specific reactions were as follows (Figure 3): using anhydrous ethanol as the solvent, EMCH and SF were refluxed with catalytic glacial acetic acid to obtain the intermediate SFZ. The C6-thiol-modified aptamer (100 μmol/L) was treated with TCEP (pH 6.5, 100 mM) at room temperature for 1 h to deprotect the thiol group. After deprotection, the aptamer was mixed with SFZ dissolved in DMF and kept at 4°C overnight. Small molecules were removed by dialysis, and the final product, the aptamer conjugate ASC, was purified via HPLC (purity > 95%). The purified product was lyophilized, resuspended and its concentration was determined for subsequent use. The structure of the key intermediate SFZ was confirmed by IR, ^1^H-NMR and mass spectrometry, while the final product ASC was characterized by mass spectrometry, HPLC and agarose gel electrophoresis. Control sequence (CRO) conjugates and Cy5-modified aptamers were synthesized using the same method (Supplementary Figures S4–S9). The calculated molecular weight of ASC was 18 918.33, and the experimentally determined value was 18 921.30.

### The stability of ASC in serum and pH-dependent drug release kinetics

**Figure 4:**
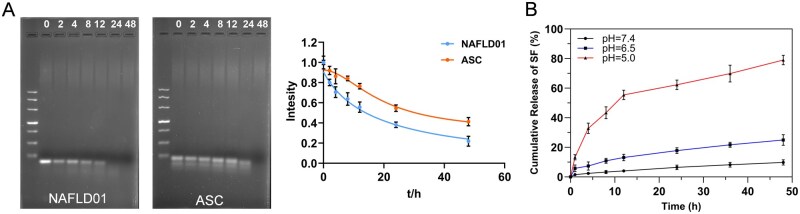
Serum stability and pH-dependent release of ASC. (**A**) Serum stability of NAFLD01 and ASC. (**B**) Cumulative ASC release at different pH values. All results are expressed as mean ± SD (*n* = 3).

To evaluate the stability of ASC in biological media, RPMI-1640 medium containing 10% FBS was prepared to simulate the growth environment. The results showed that the serum half-life of NAFLD01 was 12 h, while the ASC was 24 h, and both of them were almost degraded after 48 h. These results demonstrated that the stability of the aptamer in serum could be improved following its conjugation with SF (Figure 4A). As the structure of ASC contains a hydrazone bond, the hydrazone bond is pH sensitive [25]. The tests of drug release results showed that about 10% of SF was released from ASC at pH 7.4, while the cumulative release of SF reached approximately 25% at pH 6.5. Notably, approximately 80% of SF was released from ASC when the pH was 5.0. As the intracellular lysosomal pH is approximately 5.0, SF can be released in the acidic lysosomal environment (Figure 4B).

### ASC binding and internalization in target cells

**Figure 5:**
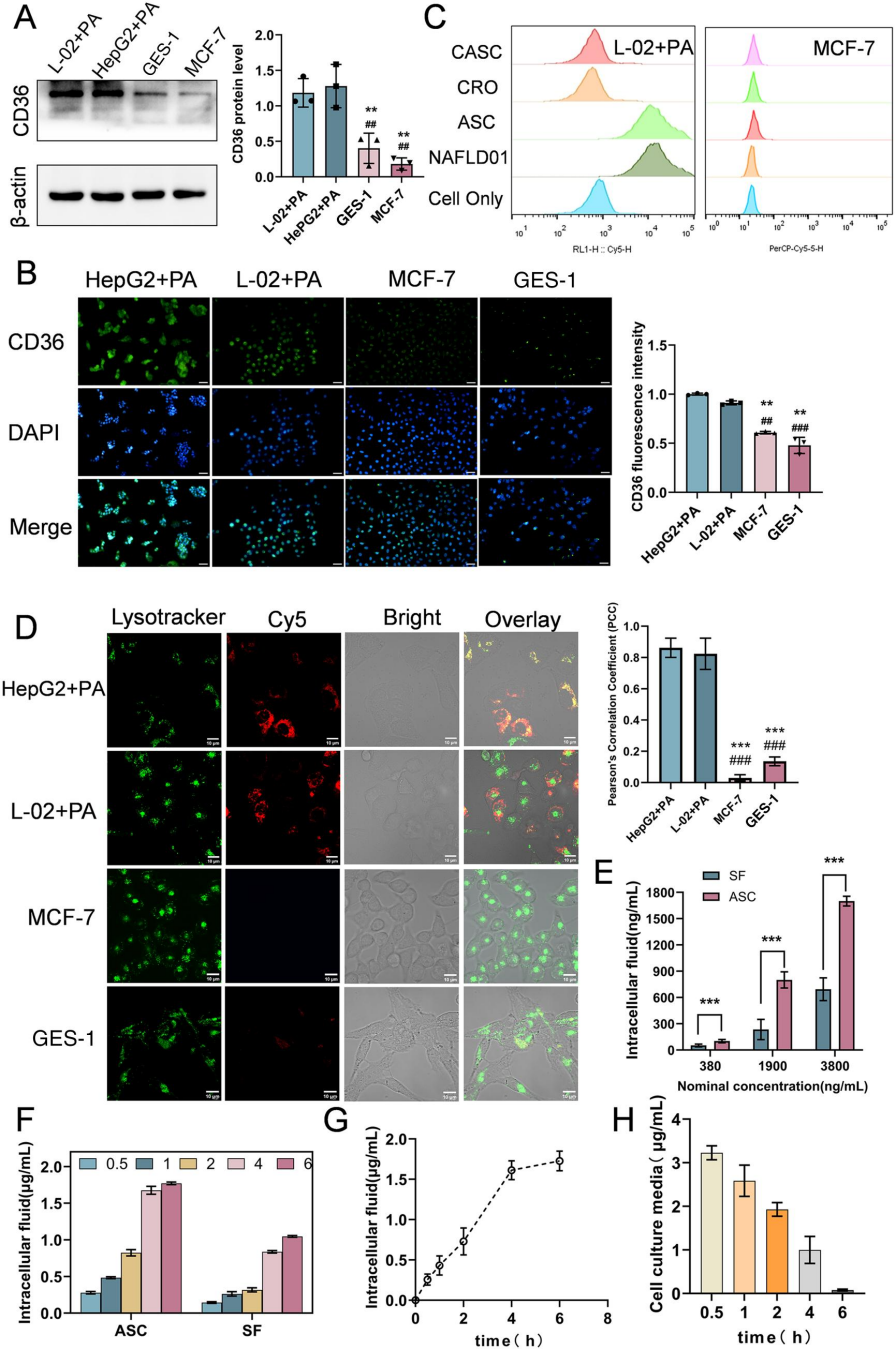
Specific binding and internalization of ASC. (**A**) Western blot analysis of CD36 protein expression. (**B**) CD36 protein expression was assessed by immunofluorescence staining. Scale bar, 20 μm. (**C**) Flow cytometric evaluation of ASC binding to L-02 and MCF-7 cells. (Cell Only: Blank cells; NAFLD01: Cy5-labeled aptamer sequence; ASC: Cy5-labeled aptamer-drug conjugate; CRO: Cy5-labeled control aptamer sequence; CASC: Cy5-labeled control aptamer sequence-drug conjugate). (**D**) Laser confocal microscopy imaging of Cy5-labeled ASC and LysoTracker-labeled lysosomes. Scale: 10 μm, observed under a 100× oil immersion objective. PCC was calculated to quantify the fluorescence co-localization between Cy5-labeled ASC and LysoTracker-labeled lysosomes. (**E**) Intracellular SF levels after treatment with free SF or ASC. (**F**) Time-dependent intracellular SF levels in L-02 cells treated with free SF or ASC. (**G**) Time-history relationship of intracellular SF concentrations of the ASC drug administration group. Observe the data for 1–6 h. (**H**) SF concentration in culture medium after SF treatment over time. Data are presented as mean ± SD (*n* = 3). ^##^*P *< 0.01, ^###^*P *< 0.001 vs. HepG2 + PA; ^**^P < 0.01, ^***^P < 0.001 vs. L-02 + PA; ^***^P < 0.001 vs. SF at the same concentration.

In this study, L-02 and HepG2 were chosen as positive cells and GES-1 and MCF-7 as negative control cells. It has been reported that exposure to PA leads to lipid deposition in hepatocytes, accompanied by a marked increase in CD36 expression. PA can up-regulate CD36 expression by activating related transcription pathways, promote fatty acid uptake and lead to lipid toxicity [26]. In addition, clinical and experimental evidence suggests that hepatic CD36 expression is increased in MASLD patients, which is closely related to lipid deposition and metabolic disorder [27]. Consistent with these results, we observed that PA treatment upregulated CD36 expression, which further supports the validity of this model for evaluating CD36-mediated targeted delivery. (Supplementary Figure S10). In contrast, the expression level of CD36 in GES-1 and MCF-7 cells was low (Figure 5A and B).

To investigate whether SF conjugation affects the specific recognition ability of the aptamer, we used Cy5-labeled ASC and its control sequence conjugate CRO‑SF (CASC) for co-incubation with cells, followed by flow cytometry analysis. The findings indicated that in modeled L‑02 cells, ASC and NAFLD01 exhibited a stronger fluorescence shift compared with CRO and CASC, whereas no detectable binding was observed in control MCF-7 cells (Figure 5C). These findings suggest that ASC retains comparable binding specificity to NAFLD01, suggesting that conjugation with SF does not significantly impair the targeting capability of the aptamer.

In order to visualize the uptake and targeting specificity of ASC in target cells, Cy5-labeled ASC was detected via laser confocal scanning microscopy, and we calculated the Pearson’s correlation coefficient (PCC) to measure the co-localization level. The results show that ASC can specifically recognize and internalize into L-02 and HepG2 cells (red fluorescence) and can be highly co-localized with the LysoTracker Green lysosomal channel (yellow fluorescence). In contrast, the weaker red fluorescence could be detected in MCF-7 and GES-1 cells (Figure 5D). We further validated efficient CD36 knockdown in PA-induced HepG2 cells via western blot (Supplementary Figure S11). High-content imaging confirmed that CD36 silencing significantly decreased ASC uptake by hepatocytes (Supplementary Figure S12). These results collectively indicated that ASC internalization is mediated by the CD36 receptor on the hepatocyte membrane [28]. The combination of NAFLD01 and SF can selectively deliver SF to target cells and enter lysosomes. According to the pH sensitivity experiment on ASC, ASC can release SF in the lysosomes of cells to exert its drug effect.

In this study, intracellular and extracellular SF levels were measured by RP-HPLC (methodological details are shown in Supplementary Figures S1 and S2; Supplementary Tables S2 and S3). Quantitative analysis was performed to compare the intracellular SF content between the ASC and the free SF group. The results showed that drugs were taken up by cells in both groups at 4 h. With increasing drug concentration, the intracellular drug concentration rose gradually, and cellular uptake was significantly greater for the ASC group compared with the free SF (Figure 5E).

In addition, the study examined the timeliness of SF uptake by cells. At 3.8 μg/mL SF, intracellular SF levels increased over time. Moreover, at different time points, intracellular SF levels were higher after ASC treatment than after free SF treatment (Figure 5F and G). At 6 h, the SF content in extracellular fluid exhibited minimal levels, which is due to degradation occurring in the media, indicating that the drug content entering the cell reached its peak at this time (Figure 5H). Collectively, these results indicate that ASC is more specific to target cells and easier to enter cells than SF.

### *In vivo* liver targeting ability of ASC

**Figure 6:**
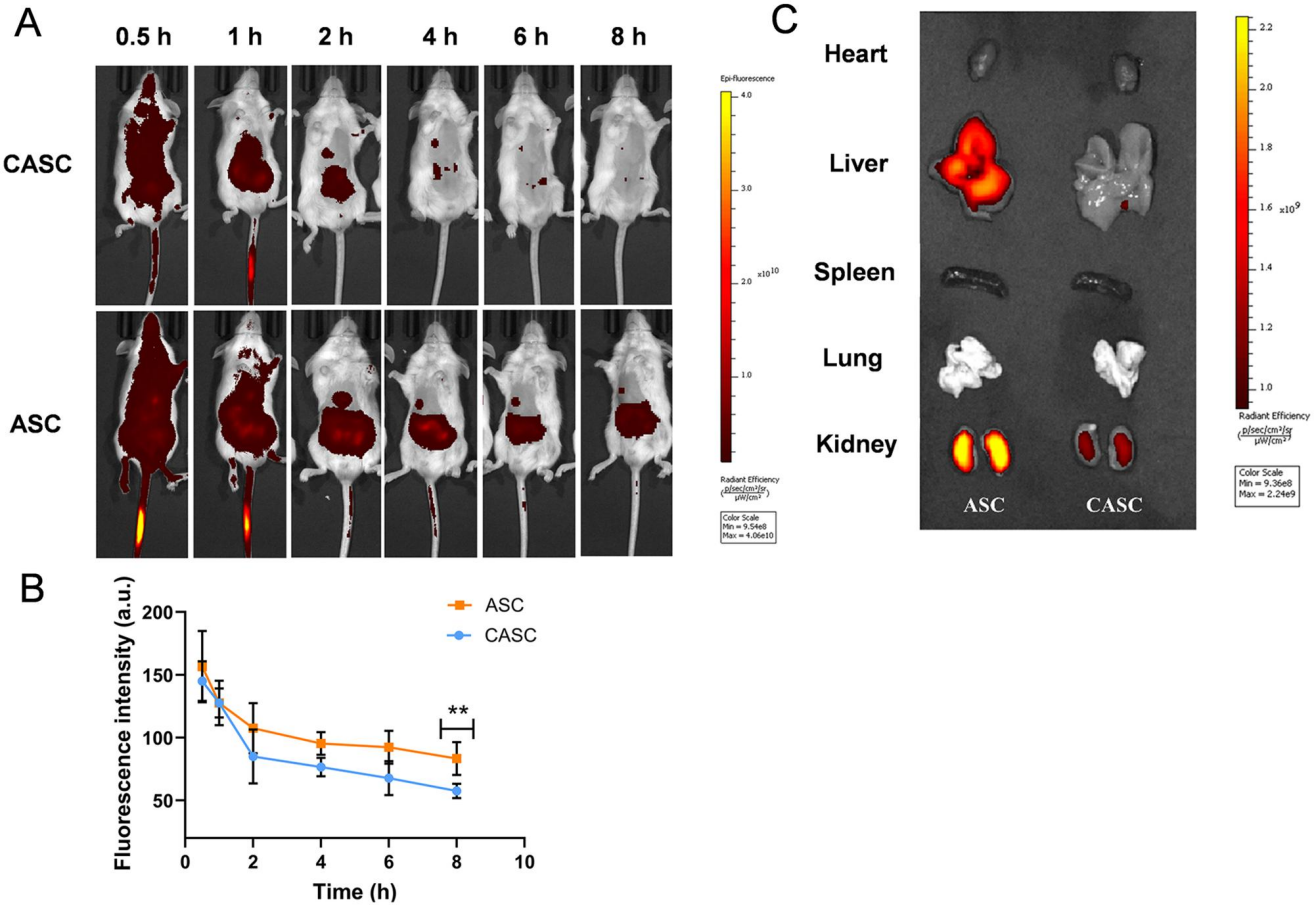
ASC liver targeting ability *in vivo*. (**A**) *In vivo* biodistribution of Cy5-labeled ASC and CASC. (**B**) *In vivo* fluorescence intensity in the liver region quantified by ROI analysis. (**C**) Organ-level distribution of Cy5-labeled ASC and CASC in major organs at 8 h after intravenous injection. ^**^P < 0.01 vs. CASC group at 8 h post-injection. Data are presented as mean ± SD (*n* = 3).

To further confirm the targeting ability of ASC *in vivo*, Cy5-ASC and Cy5-CASC were injected intravenously into MASLD mice. We performed quantitative ROI analysis on the liver region from the *in vivo* fluorescence images. The fluorescence signal in the CASC group declined rapidly over time, suggesting that CASC was rapidly metabolized *in vivo*. In contrast, ASC exhibited a significantly higher hepatic enrichment efficiency than CASC, with a robust fluorescence signal maintained in the liver at 8 h post-injection (Figure 6A and B). These findings confirmed that ASC specifically accumulated and was retained in the liver.

At 8 h after injection, the heart, liver, spleen, lung and kidney were collected for *ex vivo* fluorescence imaging of ASC and CASC. Compared with the CASC group, the ASC group showed markedly stronger hepatic Cy5 fluorescence, supporting the specific liver accumulation of ASC. Fluorescence signals were detected in the kidneys of both groups, indicating that ApDC were mainly excreted via the kidneys. In addition, no obvious fluorescent signals were observed in the heart, spleen or lung (Figure 6C).

### *In vitro* therapeutic effect evaluation of ASC

**Figure 7:**
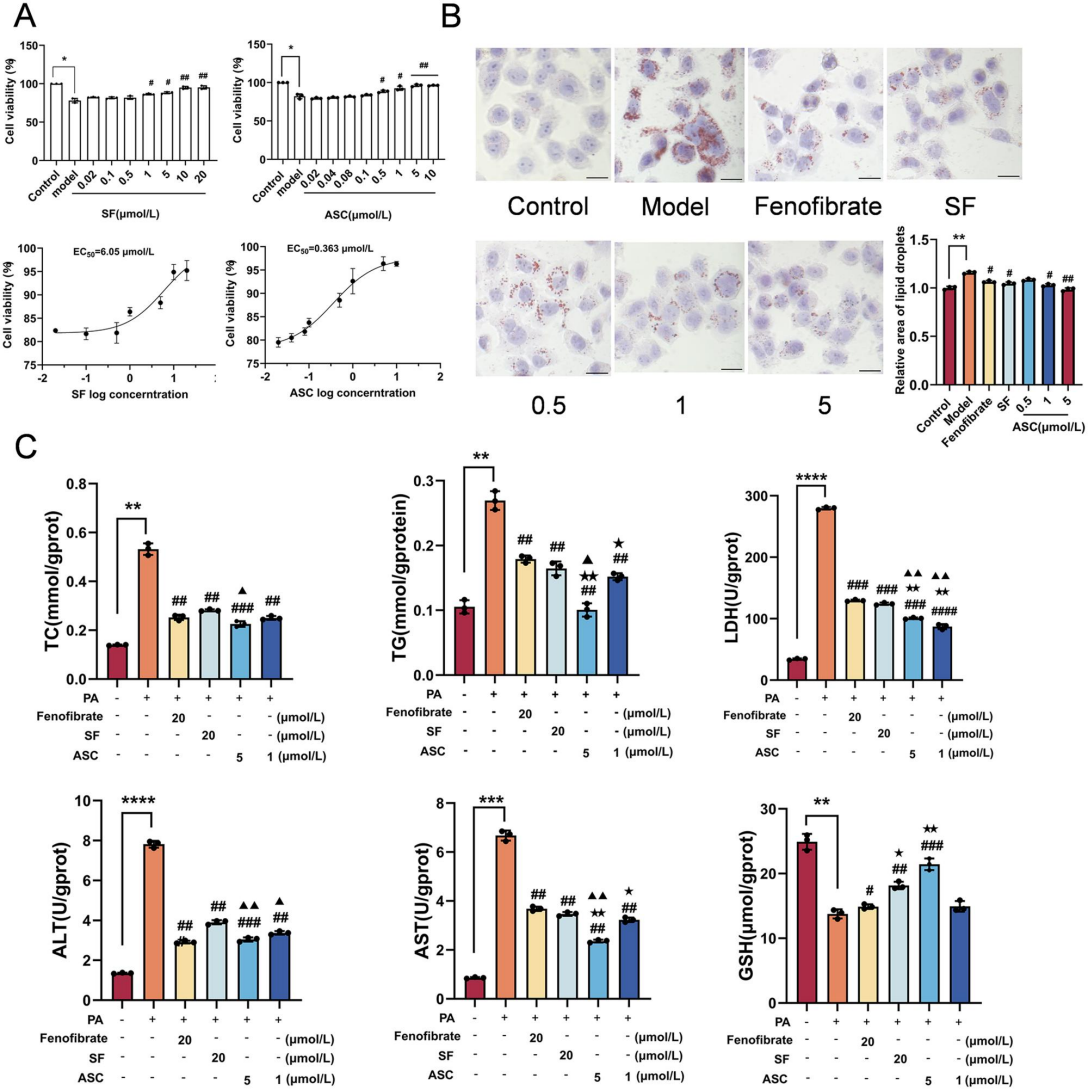
ASC reduces lipid deposition and cell injury in PA-treated L-02 cells. (**A**) L-02 cell viability after treatment with different concentrations of SF or ASC. (**B**) Oil Red O staining and quantitative analysis of intracellular lipid droplets. Scale bar, 20 μm. (**C**) Biochemical marker changes in ASC-treated L-02 cells. Data are presented as mean ± SD (*n* = 3). *P < 0.05, ^**^P < 0.01, ^***^P < 0.001 vs. control; ^#^*P *< 0.05, ^##^*P *< 0.01, ^###^*P *< 0.001 vs. model; ^★^*P *< 0.05, ^★★^*P *< 0.01 vs. fenofibrate; ^▲^*P *< 0.05, ^▲▲^*P *< 0.01 vs. SF.

To evaluate the lipid-lowering effect of ASC in L-02 cells, 500 μmol/L PA was used to induce L-02 cells to construct the MASLD cell model [29]. Model cells showed a marked reduction in viability relative to control cells, whereas it was markedly increased in the SF groups at 1, 5, 10 and 20 μmol/L and in the ASC groups at 0.5, 1, 5 and 10 μmol/L. The EC_50_ values obtained by equation fitting were 6.05 μmol/L and 0.363 μmol/L, respectively, which indicated that ASC exerted a better effect than SF (Figure 7A).

The TC, TG and Oil Red O staining results indicated marked lipid accumulation in PA-treated model cells compared with control cells (Figure 7B and C). ASC with concentrations of 1 and 5 μmol/L and SF with concentrations of 20 μmol/L could significantly improve lipid deposition, and 5 μmol/L ASC had a better therapeutic effect than SF. In addition, the indices of liver injury and oxidative stress were detected in this study, and the results indicated that following PA induction, the levels of AST, ALT and LDH in L-02 cells were significantly increased (Figure 7C), while GSH, the liver protection index, was significantly decreased. After treatment with ASC and SF, the degree of injury was reduced considerably, and the curative effect of 5 μmol/L ASC was better than that of 20 μmol/L SF and fenofibrate.

## Discussion

MASLD has emerged as a significant public health concern, with a growing disease burden and the absence of effective therapeutic interventions, thereby creating an immediate clinical demand for innovative, targeted therapeutics [30]. The clinical approval of Mylotarg for acute myeloid leukemia marked an important milestone for antibody–drug conjugates (ADCs), establishing them as a representative platform for precision therapy [31]. Similar to ADCs, ApDCs achieve targeted drug delivery through specific recognition of diseased tissues, intracellular drug release and minimized off-target toxicity [32–34]. In this study, we engineered a novel CD36-targeted ApDC by conjugating the MASLD-specific aptamer NAFLD01 with the active small molecule SF. We characterized its physicochemical properties, assessed its *in vitro* anti-MASLD activity, and investigated its liver-targeting efficiency *in vitro* and *in vivo*, with the goal of constructing a liver-targeted delivery system for MASLD.

Patients with MASLD generally require long-term medication. Therefore, a liver-targeted delivery system can greatly improve drug utilization efficiency and reduce unnecessary systemic exposure during long-term treatment [35, 36]. Network pharmacology analysis identified PPARG, PPARA and CD36 as key targets implicated in MASLD pathogenesis. Molecular docking-based screening revealed strong binding affinities between SF and these targets, suggesting that PPARG, PPARA and CD36 could serve as therapeutic targets for SF against MASLD. However, the selection of CD36 was based not only on binding affinity but also on its suitability for targeted delivery. Aptamer-mediated targeted delivery depends on the recognition of membrane-localized receptors. PPARG and PPARA belong to the nuclear receptor family and play a role in the regulation of genes related to lipid metabolism. Although these receptors play key roles in metabolic regulation, their nuclear localization limits their suitability for aptamer-mediated delivery [37]. In contrast, CD36 is a multifunctional membrane-associated protein involved in hepatic fatty acid uptake and is positively associated with disease severity in MASLD [38–40]. In addition, its membrane localization makes it readily accessible for aptamer binding and internalization. Our results showed high CD36 expression in MASLD cells, whereas only low expression was detected in normal cells, further supporting its feasibility as a selective targeted delivery receptor. Therefore, CD36 was selected as the target for developing the ApDC delivery platform in this study.

Research suggests that obese individuals exhibit a 3.5-fold higher incidence of MASLD compared with normal-weight individuals [41]. Dysfunctional adipocytes in obesity lead to elevated circulating free fatty acids, which promote hepatic triglyceride accumulation and accelerate MASLD progression [42]. In this study, MASLD was induced in mice by high-fat diet feeding [43]. Consistent with expectations, SF markedly reduced hepatic lipid accumulation in a dose-dependent manner and alleviated liver injury, as further supported by improved histopathological features. These findings indicate that SF exhibits activity against MASLD, providing a strong basis for its use in a targeted delivery system.

Based on the validated *in vivo* activity of SF, we used EMCH as a bifunctional linker to conjugate the CD36-specific aptamer NAFLD01 to SF. Notably, the acylhydrazone bond formed between EMCH and SF exhibits pH sensitivity [44]. Drug release assays demonstrated that ASC was efficiently hydrolyzed at pH 5.0, consistent with the acidic lysosomal microenvironment. This design allows ASC to remain relatively stable under physiological conditions while enabling drug release in the acidic lysosomal environment, thereby avoiding premature drug leakage.

Quantitative analysis of intracellular drug accumulation by HPLC revealed that ASC displayed significantly better targeting efficiency than free SF, a finding further supported by flow cytometry. These results indicate that the conjugation of SF with NAFLD01 does not compromise the specific targeting capability of the aptamer to CD36, while effectively enhancing intracellular SF accumulation in MASLD cells. *In vitro* assays showed that ASC exerted enhanced activity at lower doses compared with free SF and the positive control fenofibrate. No obvious cytotoxicity was observed for ASC within the therapeutic concentration range. In addition, ASC displayed superior serum stability compared with the unconjugated NAFLD01 aptamer, which may further support its *in vivo* delivery efficiency. For targeted delivery platforms, selective accumulation in diseased hepatic tissue is critical to improving therapeutic specificity and potentially reducing off-target exposure. Our *in vivo* imaging experiments indicated that ASC rapidly accumulated in the liver of MASLD mice and retained a strong fluorescent signal for at least 8 h, while the CASC was rapidly cleared with no specific hepatic accumulation. Notably, no obvious fluorescent signal accumulation was detected in the heart, lung or spleen tissues, confirming the liver-targeting specificity of the ASC platform and its potential to reduce SF distribution in non-target organs.

Although SF alone exhibited low toxicity at the tested doses, its lack of tissue specificity may still lead to unnecessary systemic exposure, particularly under long-term treatment conditions. Compared with free SF, ASC enables CD36-mediated liver-targeted delivery, enhances cellular internalization and demonstrates improved *in vitro* activity at lower doses. In addition, the pH-responsive hydrazone bond ensures specific drug release in the acidic lysosomal microenvironment. Therefore, the aptamer-mediated targeted delivery system provides ASC with higher targeting specificity, enhanced cellular uptake and more controllable drug release, which provides a foundation for the development of targeted delivery strategies for MASLD.

Some limitations remain in the present work. The therapeutic effects and safety profile of ASC in MASLD models still require further *in vivo* evaluation. Future work will concentrate on detailed pharmacodynamic and safety evaluations to further validate the therapeutic potential of ASC.

Overall, this study supports the feasibility of aptamer-mediated liver-targeted drug delivery and offers a basis for the further development of liver-targeted therapeutic strategies for MASLD.

## Conclusion

In summary, we designed a pH-responsive CD36-targeted aptamer–drug conjugate (ASC) for MASLD. The results demonstrate that ASC exhibits favorable targeting capability, controlled release behavior and enhanced intracellular delivery performance. This study supports the feasibility of aptamer-mediated liver-targeted drug delivery and offers a potential strategy for the further development of targeted therapeutic systems for MASLD.

## Supplementary Material

rbag104_Supplementary_Data

## Data Availability

Data will be made available on request.
