# Supplementary material for: A CD36-targeted aptamer-4-butyl-polyhydroxybenzophenone conjugate with pH-responsive release for liver delivery in MASLD
Source: Regen Biomater. 2026 Jun 1;13:rbag104. doi: 10.1093/rb/rbag104 (PMC13310478; doi:10.1093/rb/rbag104)
Supplement: rbag104_Supplementary_Data [file rbag104_supplementary_data.zip › 10-Jun-2026_105347_final_revised_supplemental_materials.docx]

**Supporting Information**

**A CD36-Targeted Aptamer-4-Butyl-Polyhydroxybenzophenone Conjugate with pH-Responsive Release for Liver Delivery in MASLD**

Luyao Ren^1^, Yuxi Qin^1^, Hongxiao Lu^1^, Siyu Lu^1^, Meiying Liu^1^, Huanhuan Zhang^1^, Lina Guo^1^, Guang Zhao^1^, Yunlan Li^1^*

1. School of Pharmaceutical Science, Shanxi Medical University. Taiyuan 030001, China.

*Corresponding Author: liyunlanrr@163.com.

**Materials**

1. Validation of Analytical Method
1.1. Linear relationship

According to the sample treatment method, blank cell lysis solution and blank culture solution were obtained. SF reference stock solution was added respectively, and 3 samples of SF reference intracellular fluid and 3 samples of external solution with concentrations of 0.4, 0.5, 1, 2, 3, 4, 5, 6, 7, and 8 μg/mL were prepared, and 10 μL sample was injected for HPLC analysis. The standard curves of intracellular fluid and external solution were established (Figure S1).

1.2. Specificity

Under chromatographic conditions, the chromatographic peaks of methanol solvent, SF reference substance, cell lysate, SF added to cell lysate dissolved in methanol, blank culture solution, and SF dissolved in blank culture solution were measured to investigate the specificity of the method (Figure S2).

1.3. Precision

According to the standard curve solution preparation method, SF reference cell lysate and SF reference culture solution samples with concentrations of 3.2, 4, and 4.8 μg/mL were prepared, respectively. After the sample treatment method, 10 μL was injected for HPLC analysis, and RSD% was calculated to determine the intra-day precision. One sample of SF reference cell lysate and SF reference culture solution with this concentration was prepared every day, and 10 μL was injected for HPLC analysis, and the inter-day precision of the method was calculated (Table S2).

1.4 Recovery

Prepare 1 mL of blank cell lysis solution and blank cell culture solution, weigh 3.2 mg, 4.0 mg, and 4.8 mg SF reference substance, and add them into the matrix, and dilute them to 3.2 μg/mL, 4 μg/mL, and 4.8 μg/mL, respectively, with each mass concentration in triplicate. After treatment according to the sample treatment method, inject 10 μL for HPLC analysis, and calculate the recovery rate according to the following equation (Table S3). Recovery rate =SF measured mass /SF added mass ×100%

**Abbreviations**

ADCs, antibody–drug conjugates; ALT, alanine aminotransferase; ApDC, aptamer–drug conjugate; ASC, NAFLD01–SF conjugate; AST, aspartate aminotransferase; CASC, CRO–SF conjugate; CD36, fatty acid translocase CD36; CRO, control sequence; Cy5, cyanine 5; DMF, dimethylformamide; EMCH, N-ε-maleimidocaproic acid hydrazide; GO, Gene Ontology; GSH, glutathione; H&E, hematoxylin and eosin; HCC, hepatocellular carcinoma; HDL, high-density lipoprotein; HFD, high-fat diet; HPLC, high-performance liquid chromatography; IL-1β, interleukin-1β; IL-6, interleukin-6; LDH, lactate dehydrogenase; LDL, low-density lipoprotein; MASLD, metabolic dysfunction-associated steatotic liver disease; MASH, metabolic dysfunction-associated steatohepatitis; NAFL, nonalcoholic fatty liver; NAFLD, nonalcoholic fatty liver disease; PA, palmitic acid; PCC, Pearson’s correlation coefficient; PPI, protein–protein interaction; ROI, region of interest; SELEX, systematic evolution of ligands by exponential enrichment; SF, 4-(4-(tert-butyl)benzoyl)-2,3-dihydroxyphenyl furan-2-carboxylate; TC, total cholesterol; TEAA, triethylammonium acetate; TG, triglycerides; TLC, thin-layer chromatography; TNF-α, tumor necrosis factor-α.

Table S1: Nucleic acid sequences

| Nucleic Acid Name | Sequence |
| --- | --- |
| Sulfhydryl-NAFLD01 | 5’Sulfhydryl- CGACACCTCCAGACGCACGCTCGACACGACACCTCCAGACCGCCTCGTCCACTGTGCCTC-3’ |
| Sulfhydryl-random sequence（CRO） | 5’Sulfhydryl- CCACACCTCCACACCCACCCTCCACACCACACCTCCACACCCCCTCCTCCACTCTCCCTC-3’ |
| Sulfhydryl-NAFLD01-Cy5 | 5’Sulfhydryl- CGACACCTCCAGACGCACGCTCGACACGACACCTCCAGACCGCCTCGTCCACTGTGCCTC-Cy5 3’ |
| Sulfhydryl-CRO-Cy5 | 5’Sulfhydryl- CCACACCTCCACACCCACCCTCCACACCACACCTCCACACCCCCTCCTCCACTCTCCCTC- Cy5 3’ |


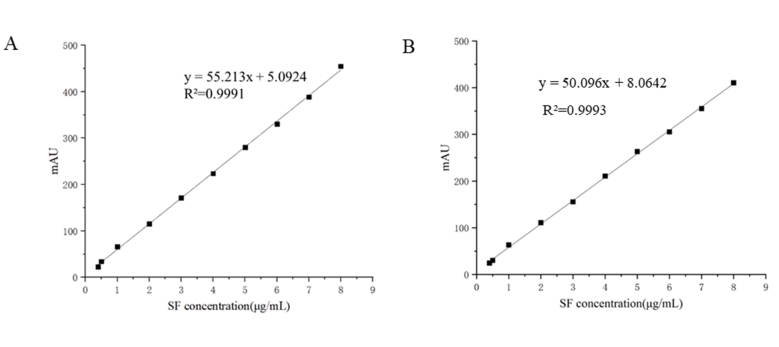


Figure S1: Standard curve (n = 3) (A): L-02 intracellular fluid SF standard curve, (B):

L-02 cell culture medium SF standard curve.


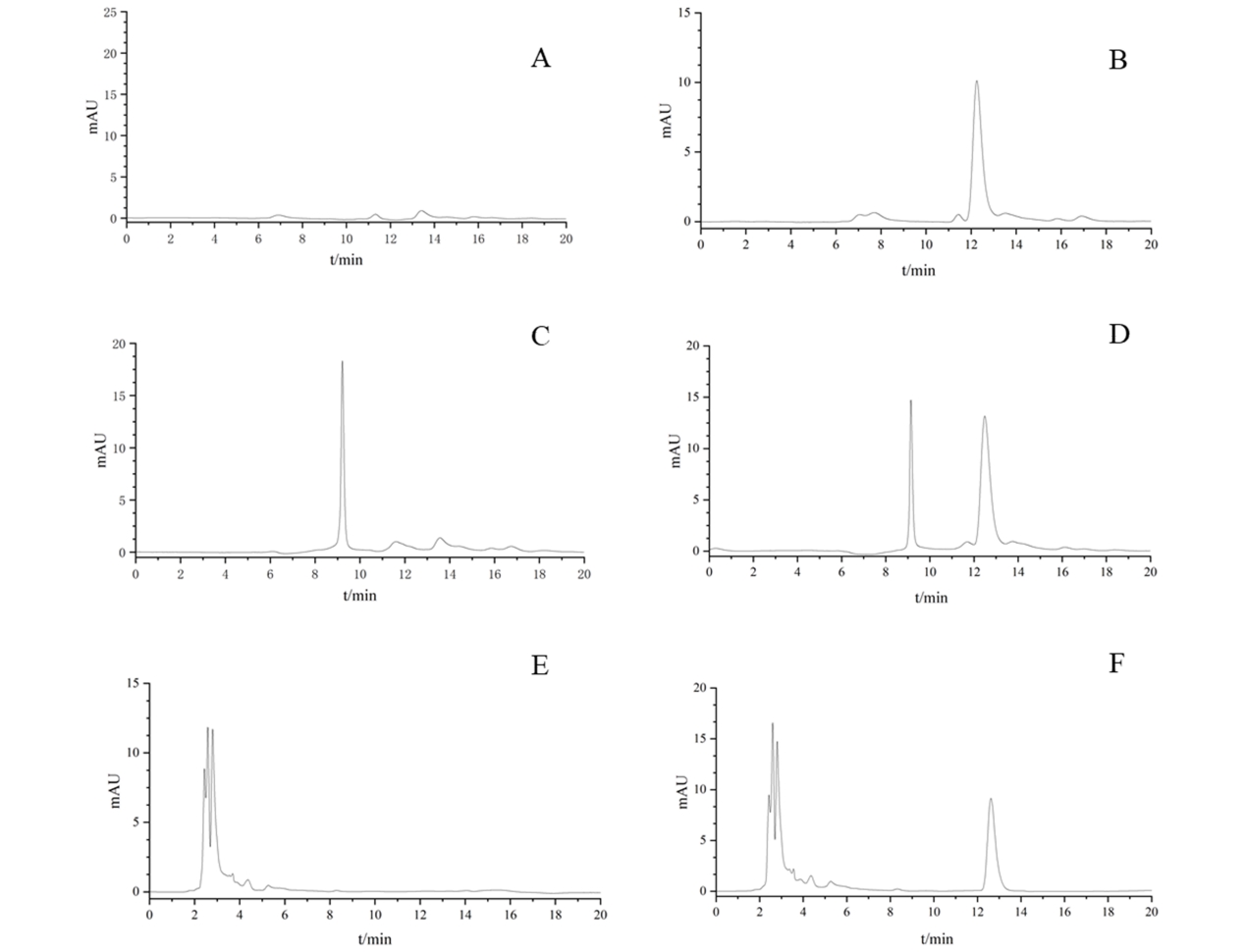


Figure S2: Chromatogram of SF standard and each sample. (A): methanol solvent, (B): SF reference substance, (C): cell lysate, (D): SF dissolved in methanol was spiked into cell lysate, (E): blank culture solution, and (F): SF was dissolved in blank culture solution.

Table S2: Precision of SF in L-02 cell lysate and cell culture medium (n=3)

| Conc.  (ng/mL) | Cell lysate | | | | | | | | | |
| --- | --- | --- | --- | --- | --- | --- | --- | --- | --- | --- |
|  | Within-Days | | | | | Between-Days | | | | |
|  | Experimental Conc.  (ng/mL) | | | Mean (ng/mL) | RSD  (%) | Experimental Conc.  (ng/mL) | | | Mean (ng/mL) | RSD  (%) |
| 3200 | 3202.2 | 3202.2 | 3118.9 | 3174.4 | 1.5 | 3135.2 | 3109.9 | 3173.3 | 3139.5 | 1.0 |
| 4000 | 3845.2 | 3959.3 | 3899.6 | 3901.4 | 1.5 | 3955.7 | 4022.7 | 3894.1 | 3957.5 | 1.6 |
| 4800 | 4649.4 | 4678.4 | 4754.4 | 4694.1 | 1.2 | 4814.2 | 4741.7 | 4709.1 | 4755.0 | 1.1 |
| Conc.  (ng/mL) | Cell culture medium | | | | | | | | | |
|  | Within-Days | | | | | Between-Days | | | | |
|  | Experimental Conc.  (ng/mL) | | | Mean (ng/mL) | RSD  (%) | Experimental Conc.  (ng/mL) | | | Mean (ng/mL) | RSD  (%) |
| 3200 | 3120.9 | 3156.0 | 3188.3 | 3155.1 | 1.1 | 3178.9 | 3137.6 | 3220.2 | 3178.9 | 1.3 |
| 4000 | 3701.0 | 3742.3 | 3762.1 | 3735.1 | 0.83 | 3764.0 | 3776.8 | 3789.6 | 3776.8 | 0.34 |
| 4800 | 4734.8 | 4729.2 | 4821.2 | 4761.7 | 1.1 | 4738.6 | 4667.2 | 4702.9 | 4702.9 | 0.76 |

Table S3: Recovery rate of SF in L-02 cell lysate and cell culture medium (n=3)

| Conc.  (μg/mL) | Cell lysate | | | | | Cell culture medium | | | | |
| --- | --- | --- | --- | --- | --- | --- | --- | --- | --- | --- |
|  | Add  (mg) | Experimental (mg) | Recovery (%) | Mean recovery (%) | RSD (%) | Add  (mg) | Experimental (mg) | Recovery (%) | Mean recovery (%) | RSD (%) |
| 3.2 | 3.2 | 3.3 | 103.1 | 101.0 | 1.8 | 3.2 | 3.2 | 100.0 | 101.1 | 1.8 |
|  | 3.2 | 3.2 | 100.0 |  |  | 3.1 | 3.1 | 100.0 |  |  |
|  | 3.2 | 3.2 | 100.0 |  |  | 3.1 | 3.2 | 103.2 |  |  |
| 4.0 | 4.0 | 3.9 | 97.5 | 99.2 | 1.5 | 4.0 | 4.0 | 100 | 98.4 | 1.4 |
|  | 4.0 | 4.0 | 100.0 |  |  | 4.1 | 4.0 | 97.6 |  |  |
|  | 3.9 | 3.9 | 100.0 |  |  | 4.0 | 3.9 | 97.5 |  |  |
| 4.8 | 4.9 | 5.1 | 104.1 | 105.6 | 1.2 | 4.8 | 4.8 | 100.0 | 100.7 | 1.2 |
|  | 4.8 | 5.1 | 106.3 |  |  | 4.7 | 4.8 | 102.1 |  |  |
|  | 4.7 | 5.0 | 106.4 |  |  | 4.8 | 4.8 | 100.0 |  |  |


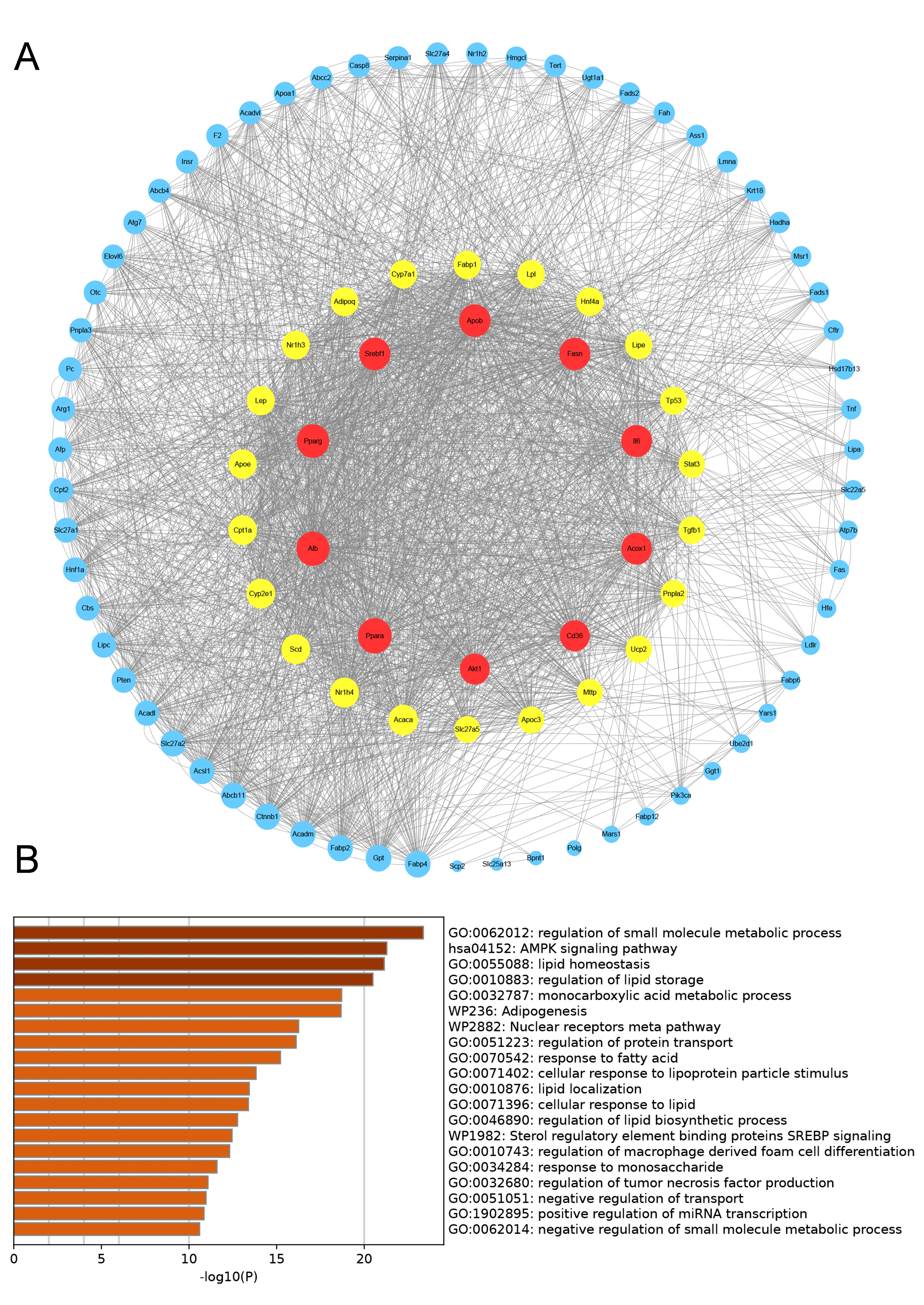


Figure S3: Bioinformatic analysis of MASLD-related targets. (A) PPI network of hub targets. (B) GO biological process enrichment of hub targets.


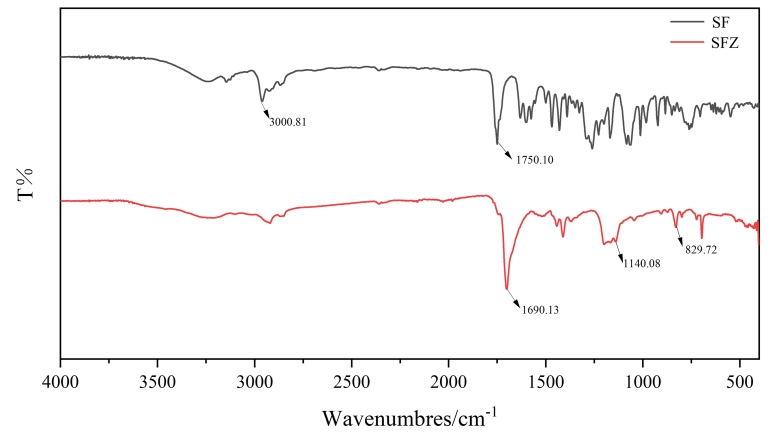


Figure S4: Infrared Spectrum of SFZ


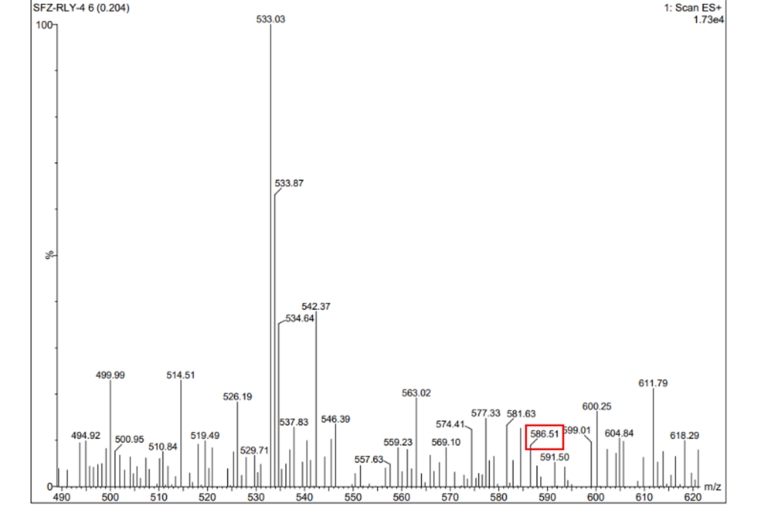


Figure S5: Mass spectrum of SFZ


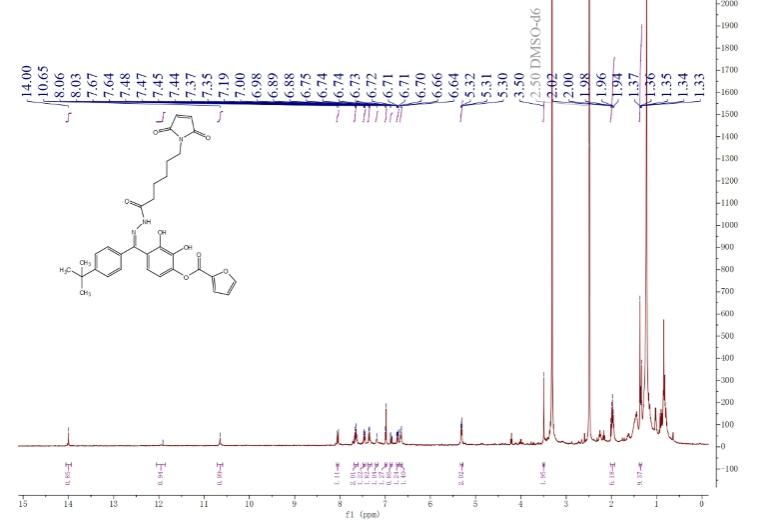


Figure S6: ^1^H-NMR of SFZ. 1H NMR (400 MHz, DMSO-d6) δ 14.00 (s, 1H), 11.91 (s, 1H), 10.65 (s, 1H), 8.05 (d, J = 9.9 Hz, 1H), 7.64 (t, J = 9.8 Hz, 2H), 7.46 (dd, J = 9.1, 3.6 Hz, 1H), 7.36 (d, J = 8.4 Hz, 2H), 7.19 (s, 1H), 6.99 (d, J = 7.3 Hz, 1H), 6.89 – 6.84 (m, 1H), 6.73 (ddd, J = 11.3, 3.7, 1.8 Hz, 1H), 6.65 (d, J = 8.9 Hz, 1H), 5.34 – 5.28 (m, 2H), 3.50 (s, 2H), 1.98 (dt, J = 13.2, 7.1 Hz, 6H), 1.39 – 1.33 (m, 9H)


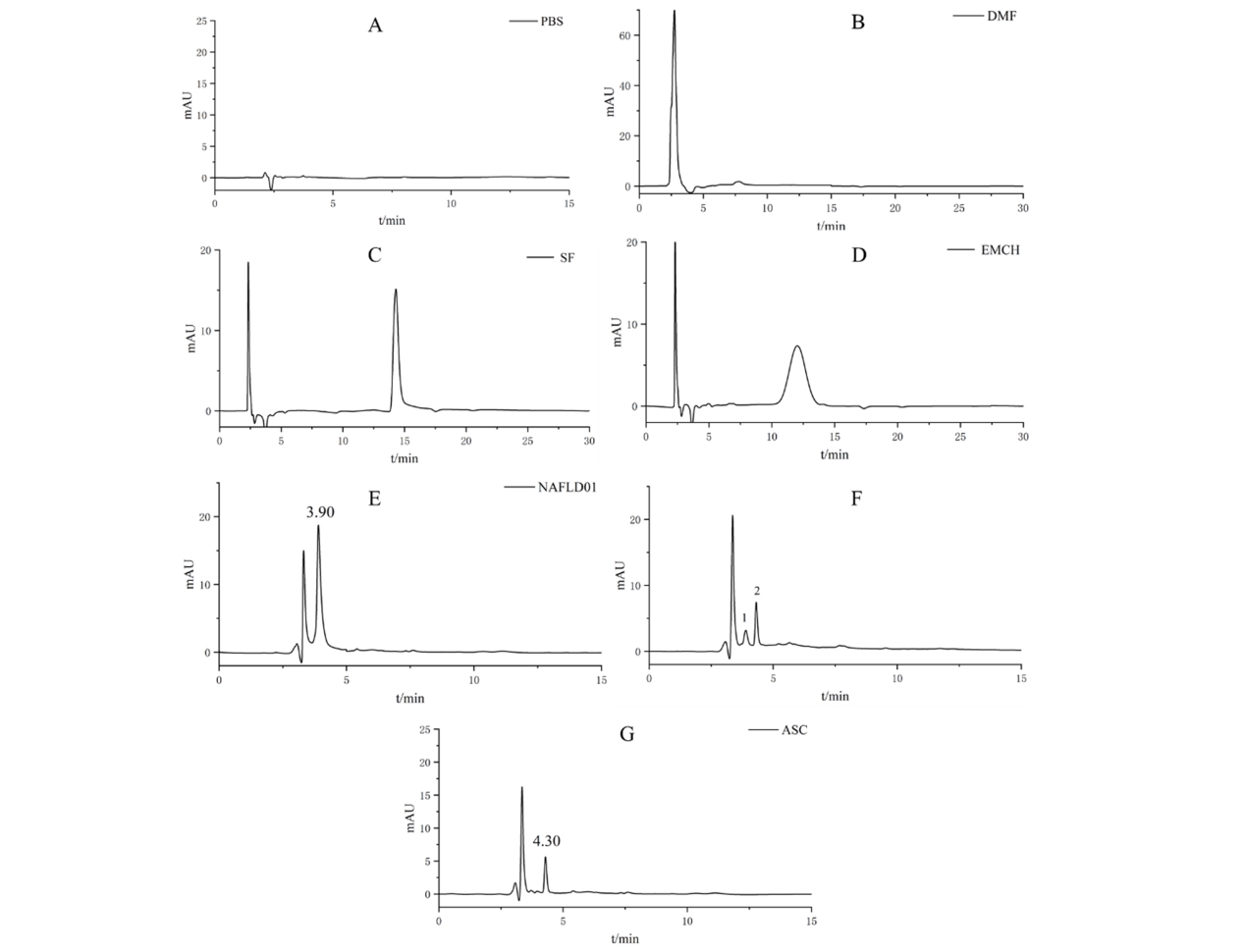


Figure S7: Representative HPLC Chromatograms of Synthetic ASC. (A): PBS; (B): DMF; (C): SF; (D): EMCH;(E): NAFLD01; (F): ASC Dialysis 12h; (G): ASC.


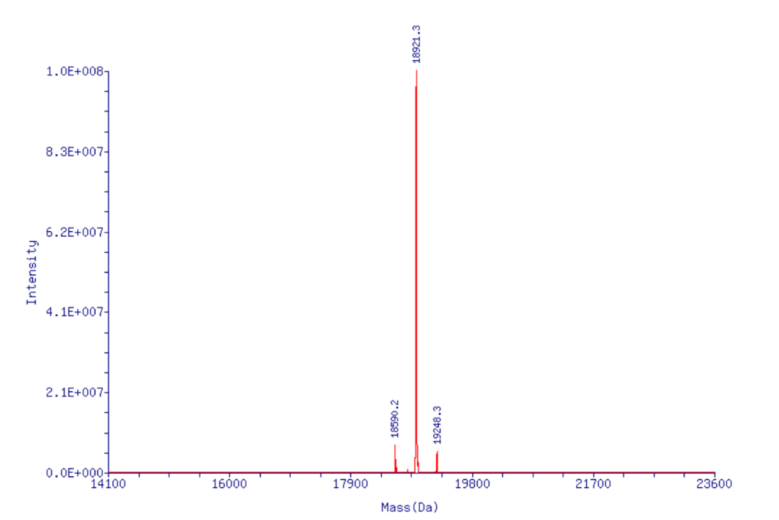


Figure S8: Mass spectrum of ASC. Calculated molecular weight: 18918.33, Found: 18921.30.


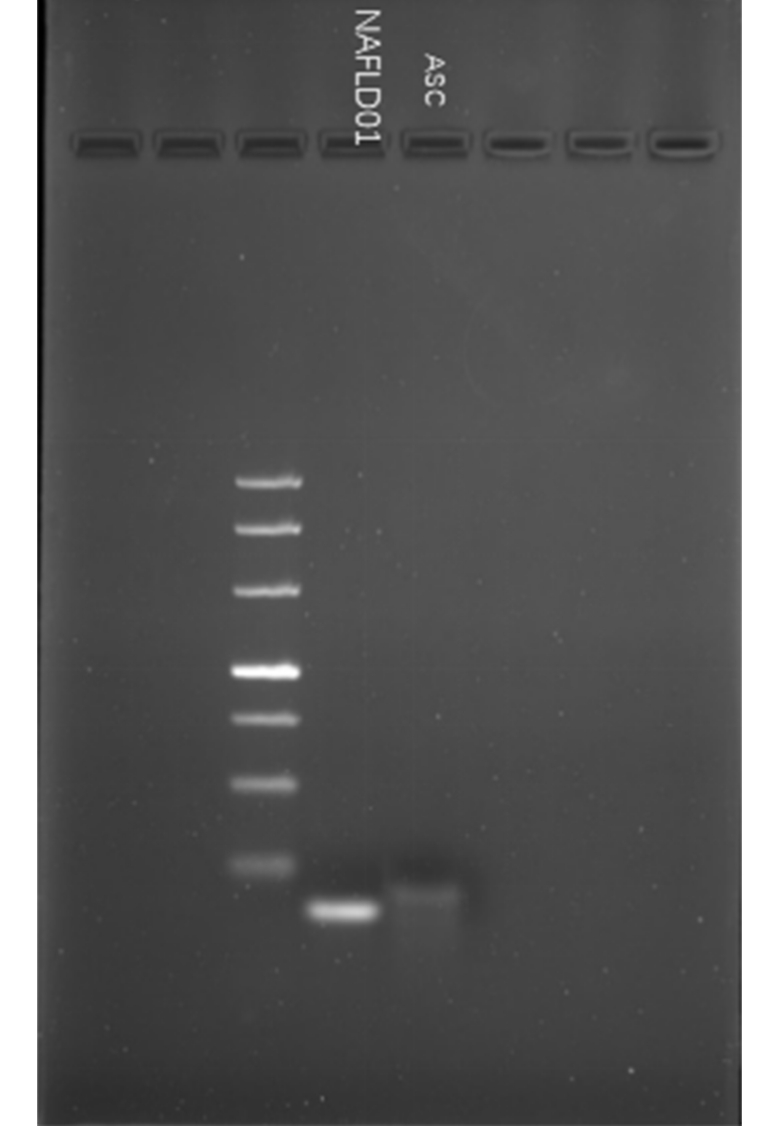


Figure S9: Agarose gel electrophoresis of NAFLD01 (left) and ASC (right).


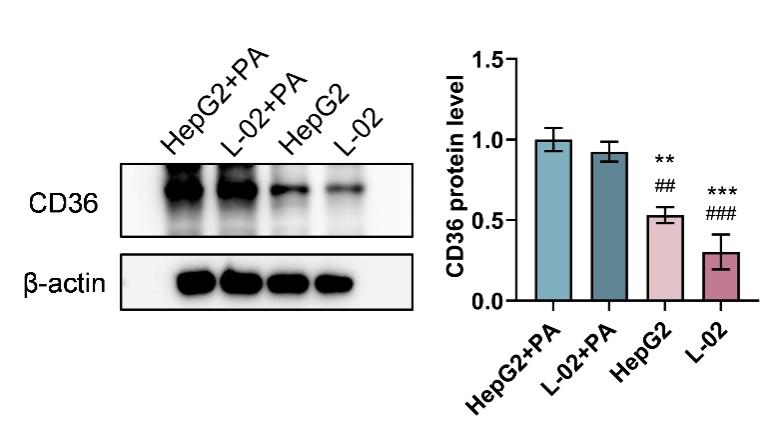


Figure S10: PA induction upregulates CD36 protein expression in MASLD model. Western blot analysis of CD36 protein expression in normal HepG2/L-02 cells and 500 μmol/L PA-induced HepG2/L-02 cells (24 h). Data are presented as mean ± SD (n = 3). ^##^*p* < 0.01, ^###^*p* < 0.001 vs. HepG2 + PA; ^**^*p* < 0.01, ^***^*p* < 0.001 vs. L-02 +PA.


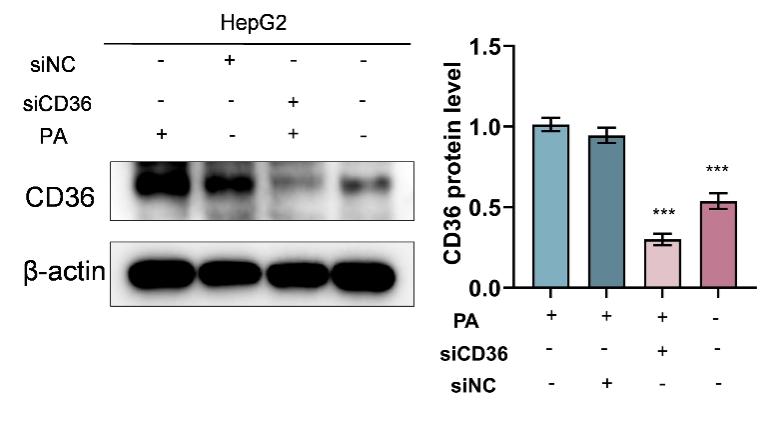


Figure S11: Validation of siCD36 knockdown efficiency in PA-induced HepG2 cells. Western blot analysis and relative quantitative results of CD36 protein expression in normal HepG2 cells, PA-induced HepG2 cells, PA-induced HepG2 cells transfected with siNC, and PA-induced HepG2 cells transfected with siCD36. Data are presented as mean ± SD (n = 3). ^***^*p* < 0.001 vs. PA-induced HepG2 cells transfected with siNC.


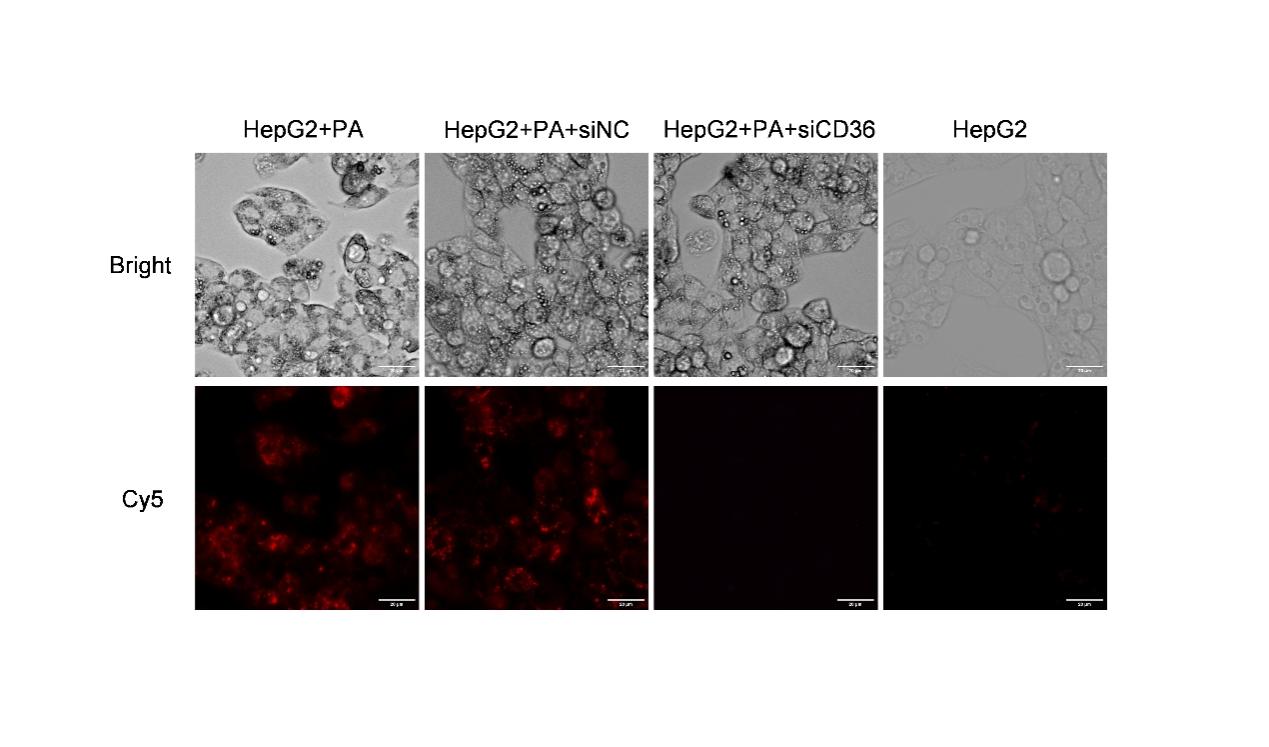


Figure S12: Effect of CD36 knockdown on ASC uptake in PA-induced HepG2 cells. High-content imaging of ASC uptake in different groups of PA-induced HepG2 cells. Cells were incubated with 250 nmol/L Cy5-labeled ASC (red fluorescence) for 2 h before image acquisition. Bright: bright field images of cell morphology; Cy5: fluorescence images of Cy5-labeled ASC, Scale: 20 μm.
